# Human umbilical cord-derived mesenchymal stem cells direct macrophage polarization to alleviate pancreatic islets dysfunction in type 2 diabetic mice

**DOI:** 10.1038/s41419-018-0801-9

**Published:** 2018-07-09

**Authors:** Yaqi Yin, Haojie Hao, Yu Cheng, Li Zang, Jiejie Liu, Jieqing Gao, Jing Xue, Zongyan Xie, Qi Zhang, Weidong Han, Yiming Mu

**Affiliations:** 1 Department of Endocrinology, Chinese PLA General Hospital, Beijing, China; 2 Department of Molecular Biology, Institute of Basic Medicine, School of Life Science, Chinese PLA General Hospital, Beijing, China; 3 Department of Geriatrics, China-Japan Friendship Hospital, Beijing, China; 4 Department of Endocrinology, Beijing Tiantan Hospital, Capital Medical University, Beijing, China

## Abstract

Progressive pancreatic β-cell dysfunction is recognized as a fundamental pathology of type 2 diabetes (T2D). Recently, mesenchymal stem cells (MSCs) have been identified in protection of islets function in T2D individuals. However, the underlying mechanisms remain elusive. It is widely accepted that β-cell dysfunction is closely related to improper accumulation of macrophages in the islets, and a series of reports suggest that MSCs possess great immunomodulatory properties by which they could elicit macrophages into an anti-inflammatory M2 state. In this study, we induced a T2D mouse model with a combination of high-fat diet (HFD) and low-dose streptozotocin (STZ), and then performed human umbilical cord-derived MSCs (hUC-MSCs) infusion to investigate whether the effect of MSCs on islets protection was related to regulation on macrophages in pancreatic islets. hUC-MSCs infusion exerted anti-diabetic effects and significantly promoted islets recovery in T2D mice. Interestingly, pancreatic inflammation was remarkably suppressed, and local M1 macrophages were directed toward an anti-inflammatory M2-like state after hUC-MSC infusion. In vitro study also proved that hUC-MSCs inhibited the activation of the M1 phenotype and induced the generation of the M2 phenotype in isolated mouse bone marrow-derived macrophages (BMDMs), peritoneal macrophages (PMs) and in THP-1 cells. Further analysis showed that M1-stimulated hUC-MSCs increased the secretion of interleukin (IL)-6, blocking which by small interfering RNA (siRNA) largely abrogated the hUC-MSCs effects on macrophages both in vitro and in vivo, resulting in dampened restoration of β-cell function and glucose homeostasis in T2D mice. In addition, MCP-1 was found to work in accordance with IL-6 in directing macrophage polarization from M1 to M2 state. These data may provide new clues for searching for the target of β-cell protection. Furthermore, hUC-MSCs may be a superior alternative in treating T2D for their macrophage polarization effects.

## Introduction

Progressive pancreatic β-cell dysfunction and apoptosis are recognized as a fundamental pathology of type 2 diabetes (T2D)^1^, and accumulated evidences suggest that part of the reason is the dramatically increased macrophages within T2D islets^2^. For example, analysis of pancreas samples from T2D patients, Goto–Kakizaki (GK) rats, db/db mice, and C57BL/6 mice fed a high-fat diet (HFD) all showed elevated numbers of macrophages within islets^3^. In addition, T2D milieu characterized by high glucose and palmitate was reported to induce secretion of chemokines from islets, which promoted the infiltration of macrophages into pancreatic islets, thus leading to progression and prolongation of islets inflammation^4,5^.

Recent studies have revealed macrophages to be quite heterogeneous^6–8^. Classically activated M1-type macrophages, elicited by Th1 cytokines alone or in concert with microbial products, play a central role in host defense by secreting pro-inflammatory cytokines such as interleukin (IL)-1β and tumor necrosis factor (TNF)-α. While alternatively activated M2-type macrophages, characterized by the expression of Fizz1, CD206, and arginase-1 (Arg1), were reported to produce anti-inflammatory cytokines and growth factors, contributing to inflammation suppression, wound healing and tissue regeneration. Eguchi and colleagues analyzed the polarity of macrophage activation within islets using flow cytometry, and found that islet-resident macrophages largely exhibited an M2-type phenotype under basal conditions^5^. However, in islets of the T2D mice, the number of macrophages dramatically increased and macrophage polarity appeared to be shifted toward M1. These M1 macrophages tended to express high levels of pro-inflammatory cytokines, subsequently resulted in progressive β-cell dysfunction and loss.

Although currently available therapeutic strategies including various oral agents and exogenous insulin can ameliorate hyperglycemia or temporarily improve insulin sensitivity, none of them can actually reverse the progressive and inexorable β-cell function damage. Mesenchymal stem cells (MSCs) are a population of fibroblast-like self-renewable cells with the potential to differentiate into multiple cell types. They can be readily isolated from a variety of adult tissues and rapidly expanded ex vivo. In recent years, umbilical cord-derived MSCs (hUC-MSCs) have been spotlighted as an appealing alternative source of stem cells due to their low immunogenicity and convenience of preparation^9^, and the clinical efficacy of hUC-MSCs in T2D patients is quite encouraging^10,11^. Results from Jianxia Hu and colleagues demonstrated that during the 36-month follow-up, infusion of hUC-MSCs significantly decreased blood glucose, glycosylated hemoglobin, and incidence of diabetic complications in T2D patients, while increasing C-peptide and homeostasis model assessment of pancreatic islet β-cell function (HOMA-β), although the precise mechanisms are yet to be elucidated^10^.

The primary mechanism by which MSCs ameliorate hyperglycemia was considered to be their potential to differentiate into insulin producing cells (IPCs), and a number of modified protocols have been applied to improve their differentiation efficacy^12,13^. Unfortunately, results in vivo were not encouraging^14,15^. Recently more attention has been paid to the immunomodulatory and anti-inflammatory effects of MSCs^16^. Some recent studies indicate that MSCs could reprogram M1 macrophages into an anti-inflammatory M2 state^17–20^. In a murine model of collagen-induced arthritis (CIA), systemically delivered hUCB-MSCs was reported to direct macrophage polarization to alleviate rheumatoid arthritis^21^. Work from our group also demonstrated that hUC-MSCs could elicit macrophages into an M2 state via secretion of IL-6 to alleviate insulin resistance in T2D rats^22^. Therefore, based on the anti-inflammatory effects of hUC-MSCs on macrophages and their therapeutic effects on islets protection^10,23,24^, we postulated that hUC-MSCs infusion could promote pancreatic islets recovery in T2D individuals via polarizing macrophages from M1 to an M2 phenotype.

In the present study, we induced a T2D mouse model and performed hUC-MSCs infusion. The polarization of macrophages within pancreatic islets was evaluated, and the possible mechanisms through which hUC-MSCs may modulate macrophages in terms of homeostatic immune balancing was explored.

## Results

### HUC-MSCs infusion improved glucose homeostasis and restored islets function in T2D mice

**Fig. 1:**
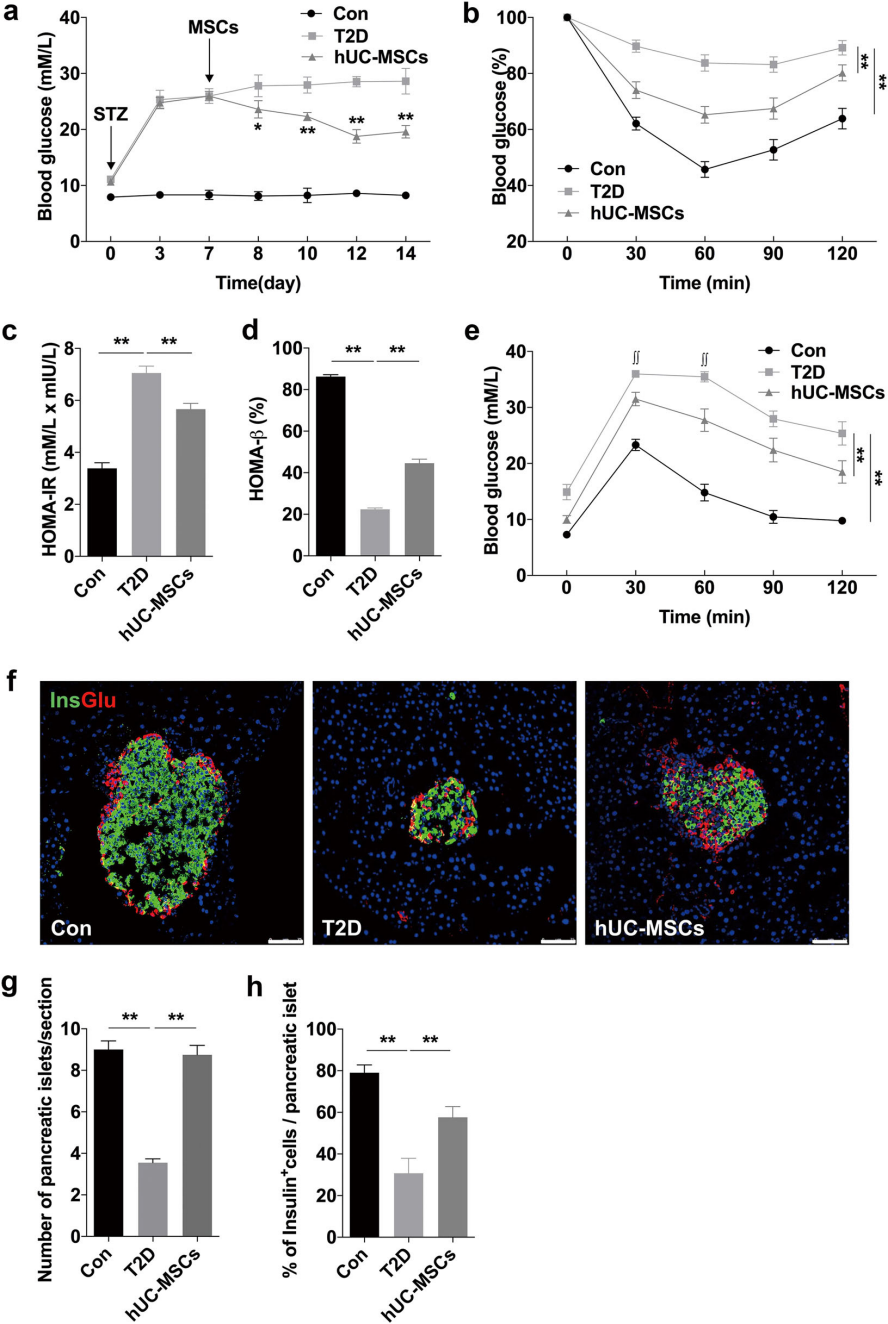
Infusion of hUC-MSCs improved glucose homeostasis in T2D mice. The T2D mice were randomly treated with an infusion of 1 × 10^6^ hUC-MSCs suspended in 0.2 ml PBS (referred as the hUC-MSCs group) or with an infusion of 0.2 ml PBS alone (referred as the T2D group). Mice fed with NCD served as control (referred as the Con group). **a** Random blood glucose level was determined consecutively after STZ injection. *p < 0.05, **p < 0.01 versus the T2D group. One week after hUC-MSCs infusion, insulin tolerance was evaluated by IPITT (**b**), results of which were presented relative to initial blood glucose levels. HOMA-IR (**c**) and HOMA-β (**d**) were calculated. Glucose tolerance was assessed by IPGTT (**e**). ^∫∫^, Blood glucose level exceeded the maximum (36 mmol/l) of the glucometer. **f** The morphology of pancreatic islets was studied by immunofluorescence according to the presence and distribution of insulin- (green) and glucagon-producing (red) cells. Scale bar, 75 μm. The number of pancreatic islets per section (**g**) and the percentage of insulin producing cells per islet (**h**) were determined by evaluating islets from at least five sections of each group. Results were presented as the means ± SD. *n* = 6 mice per group. *p < 0.05, **p < 0.01. T2D type 2 diabetes, hUC-MSCs human umbilical cord-derived mesenchymal stem cells, PBS phosphate-buffered saline, NCD normal chow diet, STZ streptozotocin, IPITT intraperitoneal insulin-tolerance tests, HOMA-IR homeostatic model assessment for insulin resistance, HOMA-β homeostasis model assessment of pancreatic islet β-cell function, IPGTT intraperitoneal glucose-tolerance tests

**Fig. 2:**
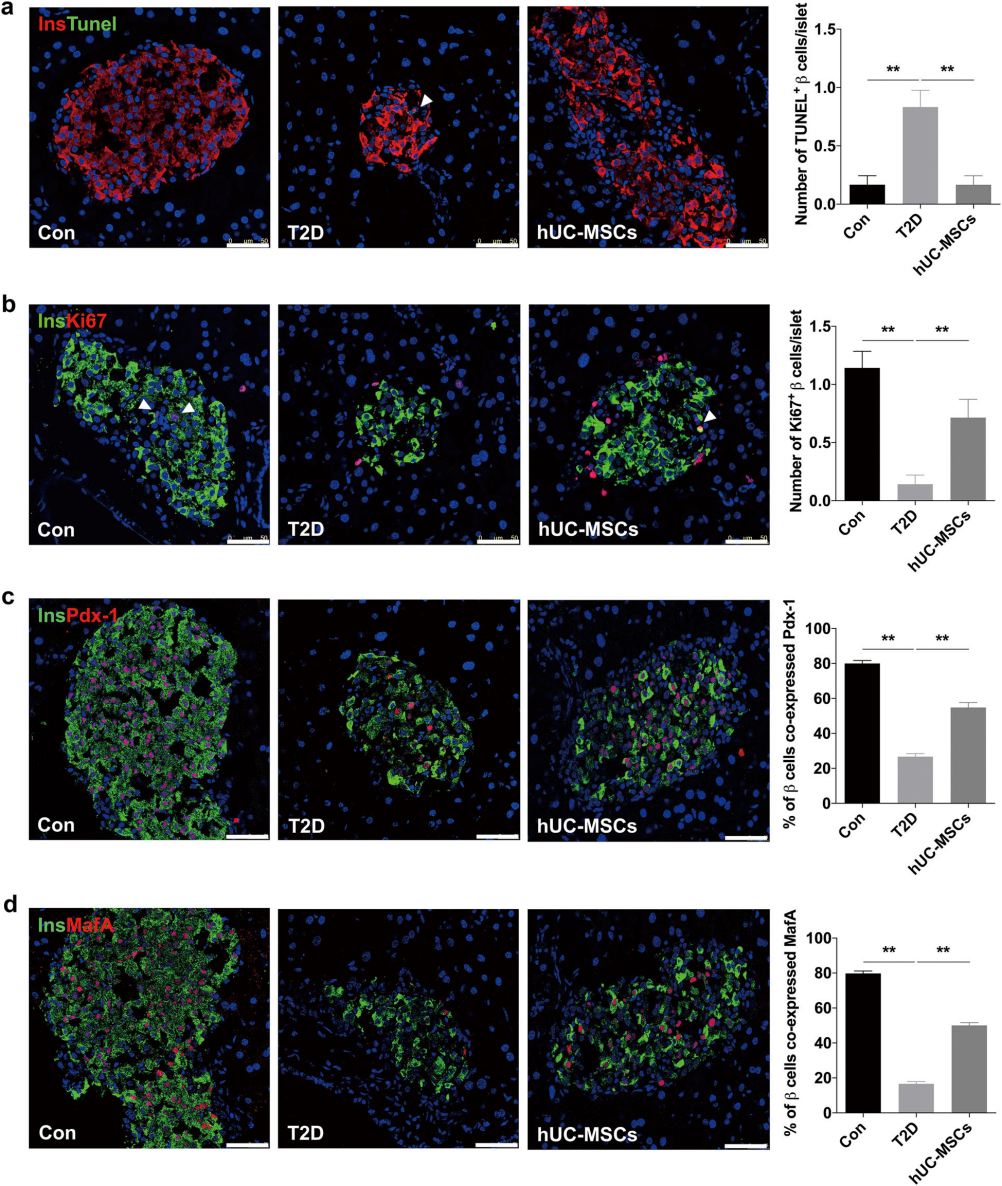
Infusion of hUC-MSCs promoted restoration of pancreatic islets function in T2D mice. **a** Apoptosis of insulin producing cells (red) was evaluated by TUNEL labeling (green), and number of TUNEL^+^ β-cells/islet was calculated. The arrow: insulin^+^ cells showing TUNEL expression. **b** Representative islets stained with antibodies against insulin (green) and Ki67 (red) of the Con, T2D and the hUC-MSCs group. Number of Ki67^+^ β-cells/islet was calculated. The arrow: insulin^+^ cells showing Ki67 expression. **c** Photomicrographs double stained with anti-insulin (green) and anti-Pdx-1 (red) antibodies of the three groups. The percentage of insulin producing cells co-expressing Pdx-1 was quantified. **d** Photomicrographs double stained with anti-insulin (green) and anti-MafA (red) antibodies of the three groups. The percentage of insulin producing cells co-expressing MafA was quantified. Scale bar, 25 μm. Quantification was determined by evaluating islets from at least 5 sections of each group. *n* = 5 mice per group. The results were presented as the means ± SD. **p < 0.01. TUNEL terminal deoxynucleotidyl transferase dUTP nick end labeling

The T2D mouse model was induced by a combination of 12-week HFD and a single introperitoneal injection of low-dose streptozotocin (STZ). The 12-week HFD added more than 10 g of weight gain compared to normal chow diet (NCD) (Supplementary Fig. 1a), and the level of random blood glucose in the HFD group was 1.22 mmol/l higher than that of the NCD group (Supplementary Fig. 1b). One week after STZ administration, blood glucose in the HFD + STZ group increased to about 26 mmol/l (Supplementary Fig. 1c). Intraperitoneal glucose tolerance tests (IPGTTs) (Supplementary Fig. 1d) and insulin-tolerance tests (IPITTs) (Supplementary Fig. 1e) showed significant deterioration of glucose disposal and insulin sensitivity in HFD + STZ treated mice, which confirmed the establishment of T2D model. The hUC-MSCs used in our study were isolated from human umbilical cords, and characteristics of them were identified as described previously^22^. As expected, hUC-MSCs infusion significantly improved glucose homeostasis in T2D mice with a gradually decreased random blood glucose level (Fig. 1a), much lower level of fasting blood glucose (FBG), and a slight increase in fasting blood insulin (FBI) (Supplementary Fig. 2). For one thing, the results of IPITT (Fig. 1b) and homeostatic model assessment for insulin resistance (HOMA-IR) (Fig. 1c) suggested a remarkable increase in insulin sensitivity after hUC-MSCs infusion, which had been deeply investigated in our previous work^22^. For another, the restoration of pancreatic β-cells was indicated by the results of HOMA-β (Fig. 1d) and IPGTT (Fig. 1e), which was the focus in this paper. Histological analysis showed that islet damage including morphological disorganization, reduction in islet size and number were markedly attenuated in the mice receiving hUC-MSCs administration compared with the non-treated T2D mice (Fig. 1f, g). Moreover, systemic hUC-MSCs infusion led to significant restoration of the ratio of β-cells per islet (Fig. 1h). We also performed insulin/TUNEL and insulin/Ki67 double immunofluorescent staining to investigate the impact of hUC-MSCs on β-cell survival and proliferation. TUNEL positive β-cells were quite rare in normal control; however, one to two apoptotic β-cells could be detected in most islets of the T2D group. After hUC-MSCs infusion, the proportion of apoptotic β-cells decreased to a level comparable with that of the normal control (Fig. 2a). Ki67 staining showed that β-cells in normal control kept a low level of proliferation rate, while after HFD and STZ injection β-cell replication was severely impaired. HUC-MSCs infusion did not elicit a robust promotion of proliferation as we expected, but a certain degree of recovery was achieved (Fig. 2b). Additionally, we evaluated the expression of two key β-cell-enriched transcription factors, Pdx1 and MafA. Immunostaining showed that the ratio of β-cells co-expressing Pdx-1 downregulated from 80.25% in normal control to <30% in the T2D group, while significantly up-regulated to 54.75 in the hUC-MSCs group, consistent with the restored β-cell function we observed (Fig. 2c). Similar results were obtained with MafA (Fig. 2d). Taken together, these data demonstrated that hUC-MSCs infusion exerted remarkable therapeutic effects against T2D. Glucose homeostasis was greatly improved and islets function in T2D individuals was markedly restored.

### HUC-MSCs infusion suppressed inflammation and induced M2 macrophage polarization in pancreatic islets

**Fig. 3:**
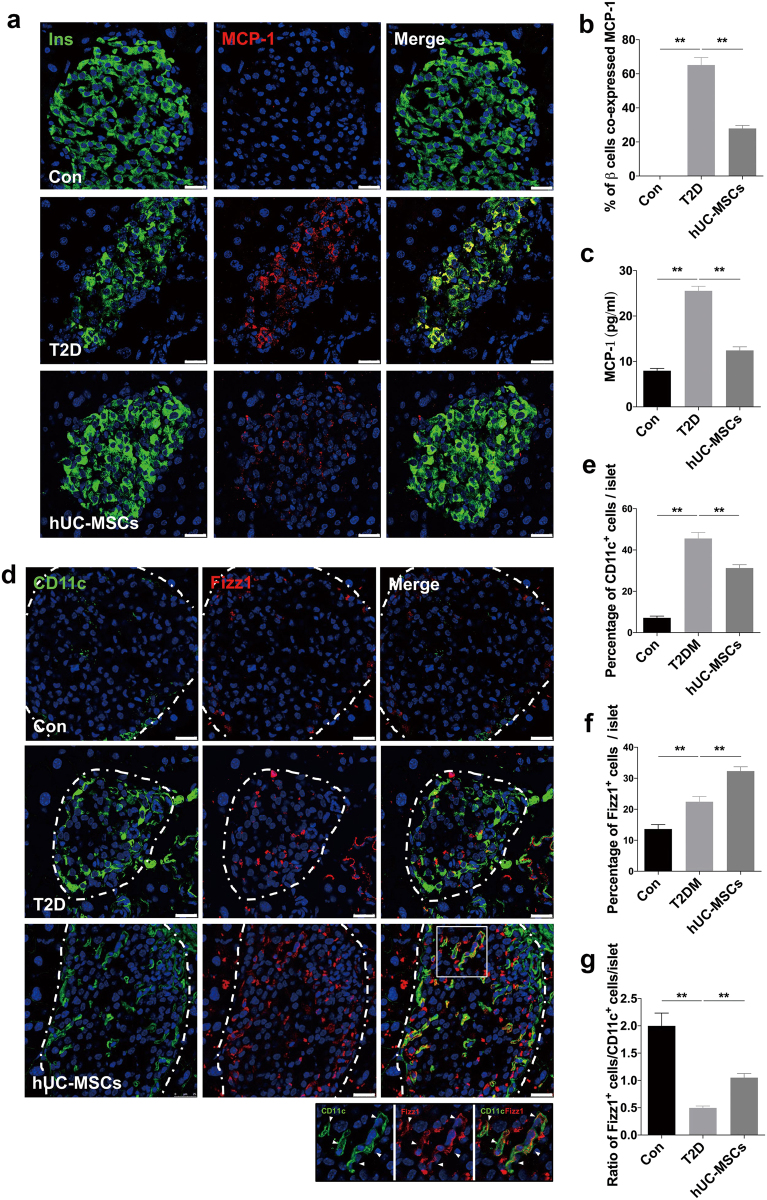
HUC-MSCs significantly downregulated the expression of MCP-1 in β-cells and induced M2 macrophage polarization in pancreatic islets. **a** Photomicrographs of representative islets stained with anti-insulin (green) and anti-MCP-1 (red) antibodies from the Con, T2D, and the hUC-MSCs group. Percentage of insulin^+^ cells expressing MCP-1was shown in **b**. **c** Serum level of MCP-1 was determined by AimPlex™ assay. **d** Photomicrographs of representative islets stained with anti-CD11c (green) and anti-Fizz1 (red) antibodies from the Con, T2D, and the hUC-MSCs group. The dotted line circled areas are pancreatic islets. The boxed region showed CD11c and Fizz1 double positive cells within islets, enlarged drawings of which were shown at the bottom of the image. Quantifications of CD11c^+^ cells and Fizz1^+^ cells within islets were separately presented in **e** and **f**. Ratio of Fizz1^+^ cells/CD11c^+^ cells was calculated and presented in **g**. Scale bar, 50 μm. *n* = 5 mice per group. The results were presented as the means ± SD. *p < 0.05, **p < 0.01. MCP-1 monocyte chemotactic protein 1

**Fig. 4:**
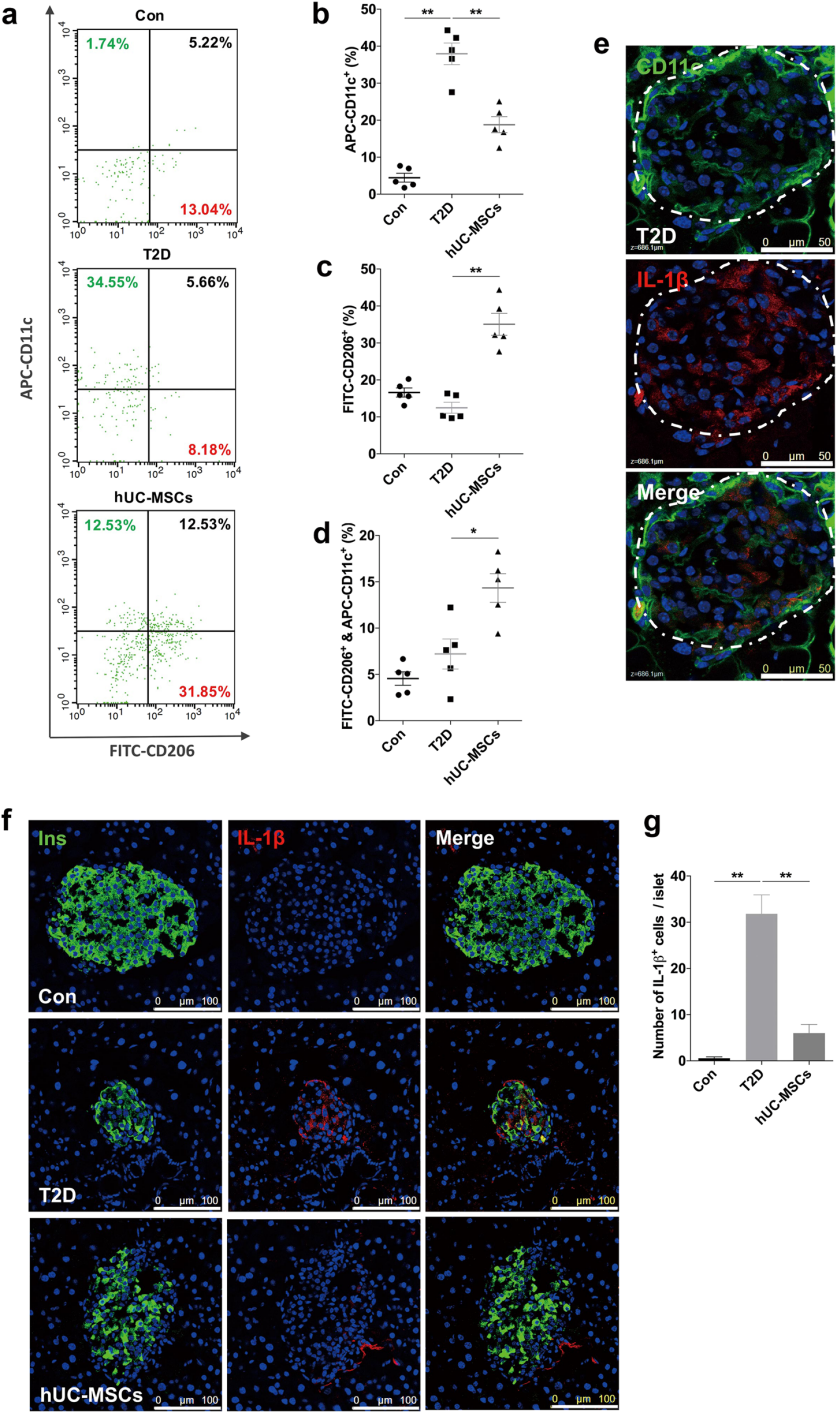
HUC-MSCs infusion induced M2 macrophage polarization and suppressed inflammation in pancreatic islets. **a** For flow cytometry analysis, freshly obtained pancreases were dissected and digested into the pellets, which were then incubated with F4/80-PE, CD11c-APC, and CD206- FITC antibodies. F4/80^+^ cells were selected for CD11c^+^ and CD206^+^ screening, proportions of which were shown in **b** and **c**, respectively. The proportion of CD11c^+^ and CD206^+^ double-positive cells were shown in **d**. **e** Photomicrographs of representative islet stained with anti-CD11c (green) and anti- IL-1β (red) antibodies from the T2D group. The dotted line circled area is pancreatic islet. Scale bar, 50 μm. **f** Photomicrographs of representative islets stained with anti-insulin (green) and anti-IL-1β (red) antibodies from the three groups. Scale bar, 100 μm. Quantification of IL-1β^+^ cells was shown in **g** and determined by evaluating islets from at least five sections of each group. PE phycoerythrin, APC allophycocyanin, FITC fluorescein isothiocyanate, TNF-α tumor necrosis factor α

Numbers of macrophages infiltrate in islets of T2D individuals, secreting multiple inflammatory cytokines, which is considered crucial for β-cell dysfunction. We thus investigated the effects of hUC-MSCs on macrophages in pancreatic islets. Since macrophage infiltration is reported to involve MCP-1 chemotaxis^25–27^, we therefore examined the expression of MCP-1 in islets. Immunostaining showed that compared with the T2D group the percentage of β-cells expressing MCP-1 was significantly downregulated after hUC-MSCs infusion (Fig. 3a, b). A similar trend was also observed in serum MCP-1 (Fig. 3c). We then used F4/80 antibody to label macrophages in islets, regardless of their sub-phenotypes. A large number of F4/80^+^ macrophages were detected in the T2D mice. However, despite of the lowered MCP-1 level, the amount of macrophages did not show much difference after UC-MSC infusion (Supplementary Fig 3a, 2b). Thus we speculated that the hUC-MSCs infusion might exert a regulatory role in the phenotypic distribution rather than the amount of macrophages. Immunofluorescence assay showed that macrophages in T2D islets were mainly CD11c (a marker for M1) positive, together with a slight increase in Fizz1^+^ (a marker for M2) cells compared with normal control. After hUC-MSCs infusion, the percentage of CD11c^+^ cells slightly reduced, while a dramatic increase of Fizz1^+^ cells within islets was observed, which led to a significant rise in the ratio of Fizz1^+^ cells to CD11c^+^ cells (Fig. 3d–g). Interestingly, CD11c/Fizz1 double-positive cells were quite noticeable in islets of the hUC-MSCs group (Fig. 3d). These results suggested that hUC-MSCs could induce M2 macrophage polarization in pancreatic islets of T2D mice, and this was further supported by the results form flow cytometry analysis for CD11c and CD206 (a marker for M2) (Fig. 4a–d) as well as the immunostaining of iNOS^+^ (a marker for M1), CD163 and ARG1 (markers for M2) (Supplementary Fig 4a-c). No significant difference was detected in the amount of pancreatic VEGF^+^ cells between the T2D and the hUC-MSCs group (Supplementary Fig 4d). Accompanied by the changes in macrophage distribution, the expression of IL-1β, which was mainly secreted by M1 macrophages (Fig. 4e), was highly expressed in T2D mice, but could only be detected in very few of the macrophages after hUC-MSCs infusion (Fig. 4f, g). Blood serum levels of IL-1β, IL-6, and TNF-α, which were upregulated after HFD and STZ administration, were downregulated by hUC-MSCs treatment (Supplementary Fig 5a-c). Serum IL-10, an anti-inflammatory molecule, was slightly increased in the hUC-MSCs group (Supplementary Fig 5d). No statistical difference was found in IL-4 (Fig. 4l) among the three groups (Supplementary Fig 5e). In summary, these data indicate that hUC-MSCs infusion could induce M2 macrophage polarization in pancreatic islets and effectively alleviate the chronic inflammation in T2D individuals.

### HUC-MSCs suppressed the activation of M1 macrophages and induced the generation of M2 macrophages in vitro

**Fig. 5:**
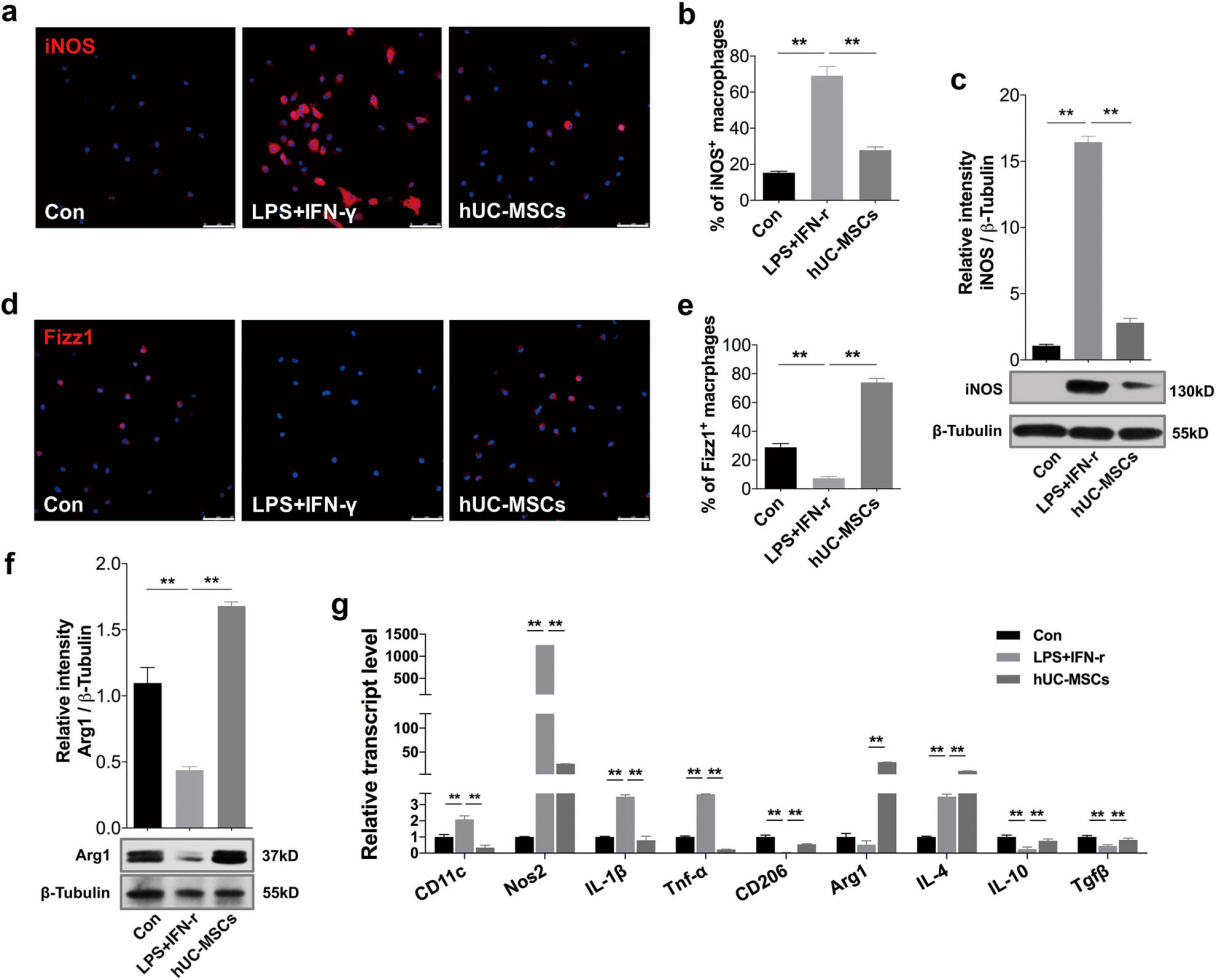
HUC-MSCs suppressed the activation of M1 macrophages and induced the generation of M2 macrophages in vitro. BMDMs and PMs were cultured alone (Con group) or in stimulation with LPS and IFN-γ in the absence (LPS + IFN-γ group) or presence of hUC-MSCs co-culturing (hUC-MSCs group). **a** Immunofluorescence of iNOS^+^-PMs in the three groups. Scale bar, 50 μm. Quantification of iNOS^+^-PMs presented in **b** was determined by evaluating at least five random fields of each section. **c** Immunoblotting analysis of iNOS in BMDMs. Relative protein level is quantified by ratio of iNOS to β-tubulin. **d** Immunofluorescence of Fizz1^+^-PMs in the three groups. Scale bar, 50 μm. Quantification of Fizz1^+^ PMs presented in **e** was determined by evaluating at least five random fields of each section. **f** Immunoblotting analysis of Arg1 in BMDMs. Relative protein level is quantified by ratio of Arg1 to β-tubulin. **g** Quantitative RT-PCR analysis of gene expression in BMDMs from the three groups. Results are presented relative to those of the control group, set as 1. Values are means ± SD of three individual experiments, **p < 0.01. BMDMs bone marrow-derived macrophages, PMs peritoneal macrophages, LPS lipopolysaccharides, IFN- γ interferon- γ, iNOS inducible nitric oxide synthase, Arg1 arginase-1, RT-PCR reverse transcriptase polymerase chain reaction, Nos2 nitric oxide synthase 2, Tgfβ transforming growth factor-β

Next, we sought to confirm the modulatory effects of hUC-MSCs on the phenotype of macrophages in vitro. Mouse bone marrow-derived macrophages (BMDMs) and peritoneal macrophages (PMs) were isolated. Flow cytometry analyses showed that the positive rate of F4/80 in the bone marrow-derived cells was over 95% (Supplementary Fig. 6a). And immunofluorescence detection revealed that about 93% of the cells achieved by peritoneal lavage were F4/80 positive (Supplementary Fig. 7b). Lipopolysaccharides (LPS) and interferon-γ (IFN-γ) were used to induce BMDMs and PMs to polarize towards M1 phenotype. The stimulated BMDMs and PMs were then co-cultured with hUC-MSCs in a trans-well system for 24 h. In comparison with the LPS- and IFN-γ-stimulated group, the expression of iNOS was significantly down-regulated in the hUC-MSCs co-cultured group (Fig. 5a–c). Results of immunofluorescence from PMs (Fig. 5a, b), and immunoblotting (Fig. 5c) from BMDMs all supported this trend. Meanwhile, the protein expression of Fizz1 (in PMs) (Fig. 5d, e) and Arg1 (in BMDMs) (Fig. 5f), markers for M2 macrophages, were significantly upregulated after hUC-MSCs co-culture. Consistently, quantitative RT-PCR analysis revealed higher expression of genes encoding M2 macrophages (CD206 and Arg1) and anti-inflammatory mediators (IL-4, IL-10, and Tgfβ) in the hUC-MSCs co-cultured BMDMs compared with the LPS- and IFN-γ-stimulated BMDMs, while lower expression of genes encoding M1 macrophages (CD11c, Nos2) and pro-inflammatory molecules (IL-1β and TNF-α) (Fig. 5g). Moreover, the secretion of cytokines in the supernatant of BMDMs were measured by AimPlex multiplex assay, results of which showed that the levels of IL-1β (Supplementary Fig 7a) and TNF-α (Supplementary Fig 7b) significantly decreased after hUC-MSCs incubation, and the level of IL-4 increased (Supplementary Fig 7c). Additionally, to address the issue that hUC-MSCs could influence to “human” macrophages, THP-1 cells was stimulated by LPS and IFN-γ (Supplementary Fig 8) or a combination of cytokines including IAPP, CCL2, CXCL1, and IL-1β (to mimic microenvironment of T2D pancreas) (Supplementary Fig 9) and then co-cultured with hUC-MSCs. The results from RT-PCR, ELISA, immunofluorescent staining, and cytometry analysis demonstrated that hUC-MSCs could inhibit classical M1 activation and convert macrophages into an anti-inflammatory M2 phenotype through paracrine mechanism.

### HUC-MSCs modulated macrophage polarization via secretion of IL-6

**Fig. 6:**
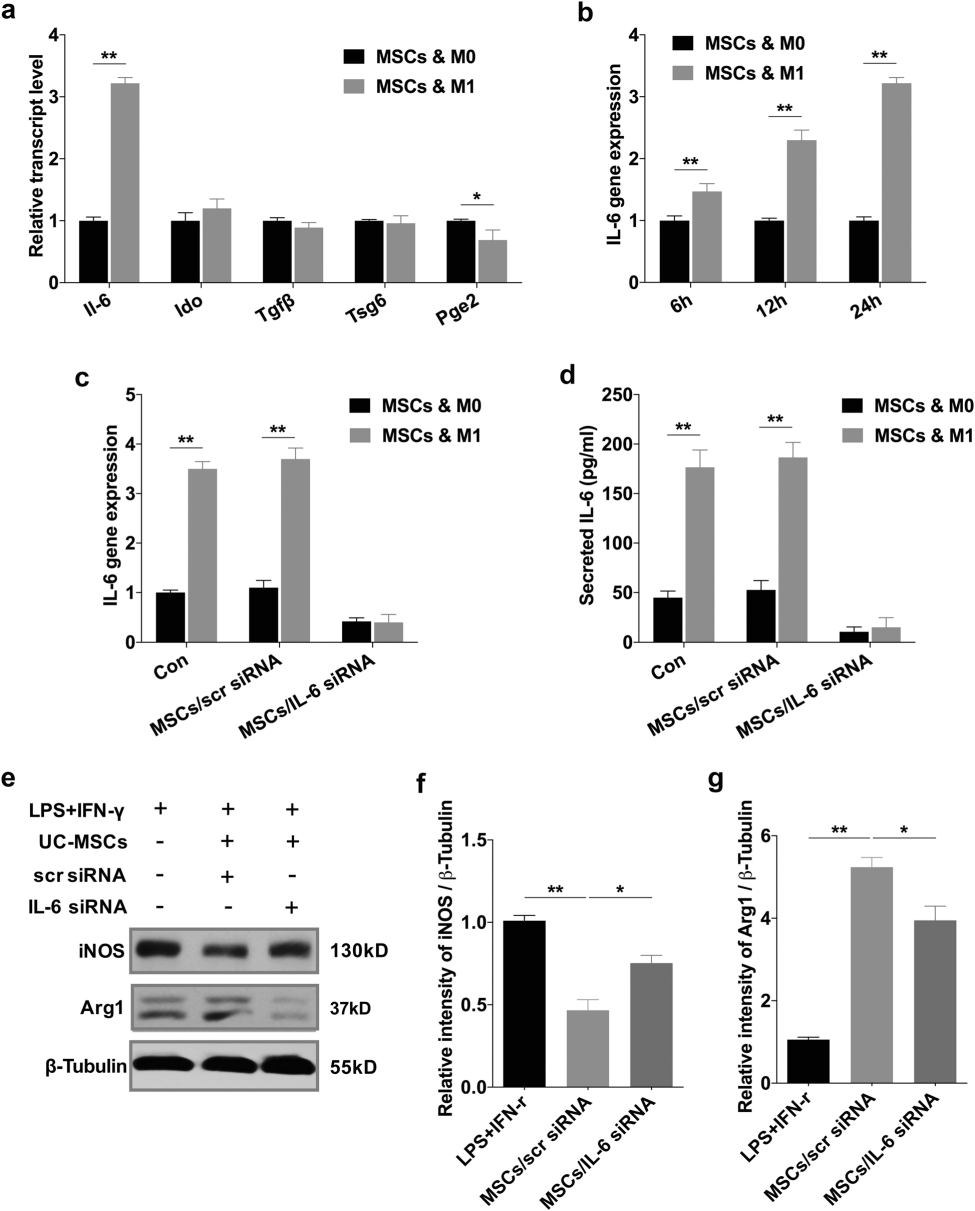
HUC-MSCs modulated macrophage polarization via secretion of IL-6 in vitro. **a** Relative gene expression levels of immunomodulatory factors involve in polarization modulatory effect of hUC-MSCs on macrophages. hUC-MSCs were cultured with primary BMDMs (M0) or with LPS- and IFN-γ-stimulated BMDMs (M1) for 24 h. Results are presented relative to those of hUC-MSCs cultured with M0, set as 1. **b** Quantitative RT-PCR analysis of IL-6 expression in hUC-MSCs. HUC-MSCs were cultured with M0 or M1 for 6, 12, 24 h, results are presented relative to those of hUC-MSCs cultured with M0, set as 1. **c** Quantitative RT-PCR analysis of IL-6 expression in hUC-MSCs (Con), hUC-MSCs transfected with scrambled siRNA (MSCs/scr siRNA), or hUC-MSCs with IL-6 siRNA (MSCs/IL-6 siRNA). HUC-MSCs were cultured with M0 or with M1. **d** Enzyme-linked immunosorbent assays (ELISA) of IL-6 in medium of hUC-MSCs cultured with M0 or M1, symbols are as in **c**. **e** Immunoblotting analysis of BMDMs in stimulation with LPS and IFN-γ cultured alone, cultured with hUC-MSCs transfected with scr siRNA, or with hUC-MSCs transfected with IL-6 siRNA. Relative protein level is quantified by ratio of iNOS to β-tubulin and that of Arg1 to β-tubulin, and separately shown in **f** and **g**. Values are means ± SD of three individual experiments, *p < 0.05, **p < 0.01. IL-6 interleukin 6, Ido indoleamine 2, 3-dioxygenase. Tsg6 TNF-α-induced protein 6, Pge2 prostaglandin E2

**Fig. 7:**
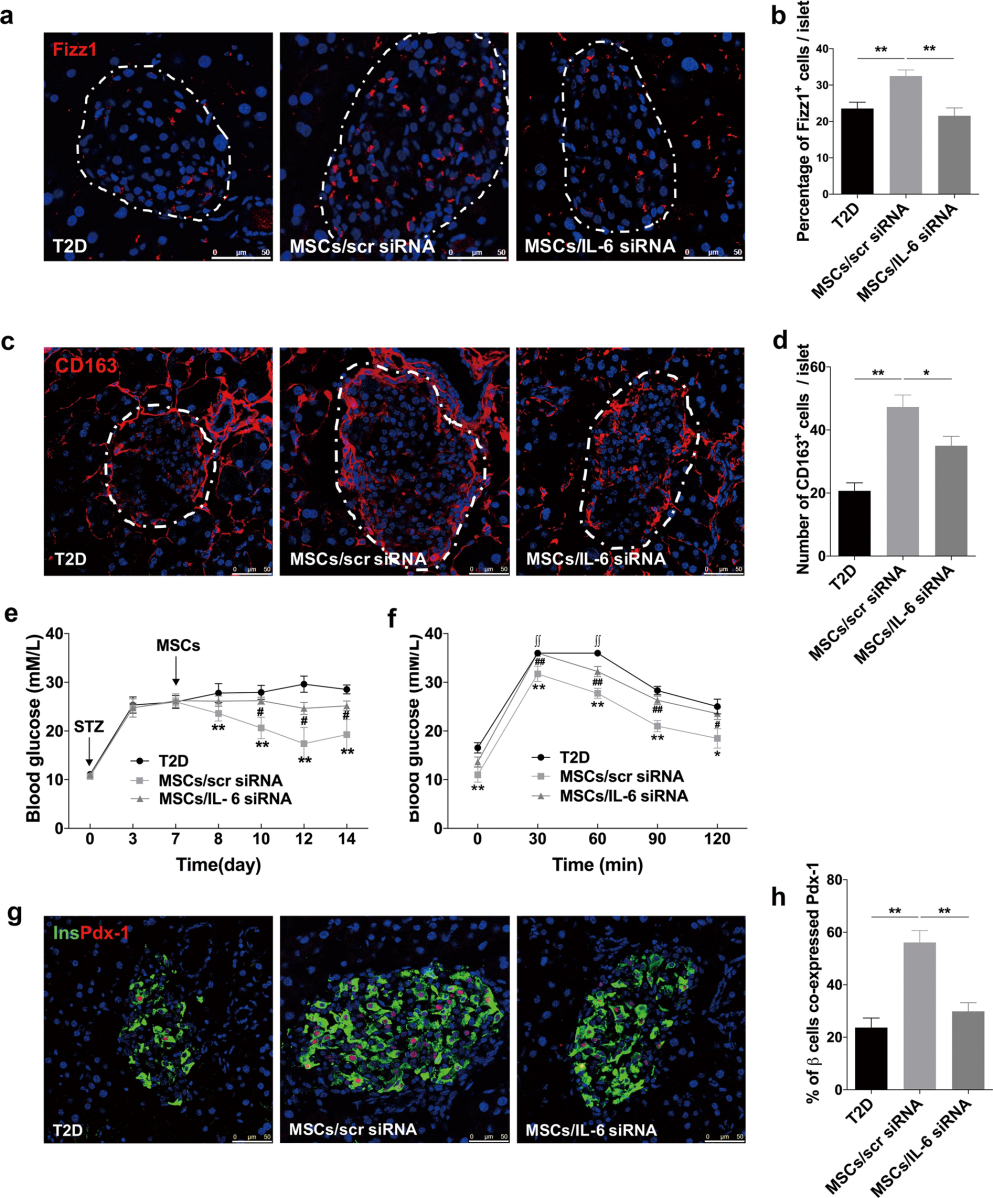
HUC-MSCs modulated macrophage polarization via secretion of IL-6 in vivo. Photomicrographs of representative islets stained with anti-Fizz1 (red) (**a**) and anti-CD163 (red) (**c**) antibodies from the T2D mice without (T2D group) or with the administration of hUC-MSCs transfected with scrambled siRNA (MSCs/scr siRNA group) or hUC-MSCs transfected with IL-6 siRNA (MSCs/IL-6 siRNA group). The dotted line circled areas are pancreatic islets. Quantifications of Fizz1^+^ cells and CD163^+^ cells within islets were determined by evaluating islets from at least five sections of each group, results of which were separately presented in **b** and **d**. **e** Blood glucose level was determined consecutively after STZ injection. Values are means ± SD. **p < 0.01 for the MSCs/scr siRNA group versus the T2D group. ^**#**^*p* < 0.05 for MSCs/IL-6 siRNA group versus the MSCs/scr siRNA group. **f** Glucose tolerance was assessed by IPGTT. ∫∫, blood glucose level exceeded the maximum (36 mmol/l) of the glucometer. **g** Photomicrographs double stained with anti-insulin (green) and anti-Pdx-1 (red) antibodies of the three groups. The percentage of insulin-producing cells co-expressing Pdx-1 was quantified by evaluating islets from at least five sections of each group, results of which were shown in **h**. Scale bar, 50 μm. *n* = 5 mice per group. The results were presented as the means ± SD. *p < 0.05, **p < 0.01

Given that immunomodulatory effects of MSCs are reported to be non-instinctive but rather initiated by inflammatory mediators^28–30^, we hypothesized that the disease-specific inflammatory microenvironment may affect the regulatory effects of hUC-MSCs. Therefore, we evaluated the expression changes of several crucial immunomodulatory factors in hUC-MSCs after they were co-cultured with M1 BMDMs. These immunomodulatory factors include IL-6, indoleamine 2, 3-dioxygenase (Ido), transforming growth factor-β (Tgfβ), TNF-α-induced protein 6 (Tsg6), and prostaglandin E2 (Pge2), which are reported to involve in polarization modulatory effects of MSCs on macrophages^16,22,31^. The results showed that no significant difference was detected in the expression of Ido, Tgfβ, and Tsg6. A slight decrease was observed in the gene encoding Pge2, whereas only the expression of IL-6 was significantly increased (Fig. 6a). Further research showed that IL-6 expression in hUC-MSCs gradually and continuously upregulated within 24-h incubation (Fig. 6b, c), and the secretion level of IL-6 in the supernatants increased to as high as 176.6 pg/ml (Fig. 6d). In vivo, IL-6 and hUC-MSCs co-staining was also observed in hUC-MSCs-treated T2D mice (Supplementary Fig 10). We thus inhibited IL-6 in hUC-MSCs using small interfering RNA (siRNA). Transcription (Fig. 6c) and secretion (Fig. 6d) levels of IL-6 were significantly reduced by IL-6 siRNA. The results of immunoblotting analysis revealed that hUC-MSCs transfected with the scrambled siRNA (scr siRNA) significantly decreased the expression of iNOS, and increased that of Arg1 in BMDMs, whereas the effect of hUC-MSCs transfected with IL-6 siRNA on regulating the expression of iNOS and Arg1 was partially abrogated (Fig. 6e–g). Consistently, our in vivo study on Fizz1^+^ cells (Fig. 7a, b), CD163^+^ cells (Fig. 7c, d) and CD11c^+^ cells (Supplementary Fig 11a, b) revealed that the effect of hUC-MSCs on modulating macrophage phenotype in pancreatic islets was also dampened after transfected with IL-6 siRNA. As expected, the therapeutic effects of hUC-MSCs on glucose homeostasis and islets protection were impaired, which were indicated by the results of random blood glucose (Fig. 7e), IPGTT (Fig. 7f), and expression of PDX-1 in β-cells (Fig. 7g, h). All together, these data demonstrated that hUC-MSCs regulate macrophage polarization at least partially through IL-6 secretion, resulting in restoration of β-cell function and glucose homeostasis.

### HUC-MSCs secreted MCP-1 to coordinate with IL-6 in regulating macrophage polarization

**Fig. 8:**
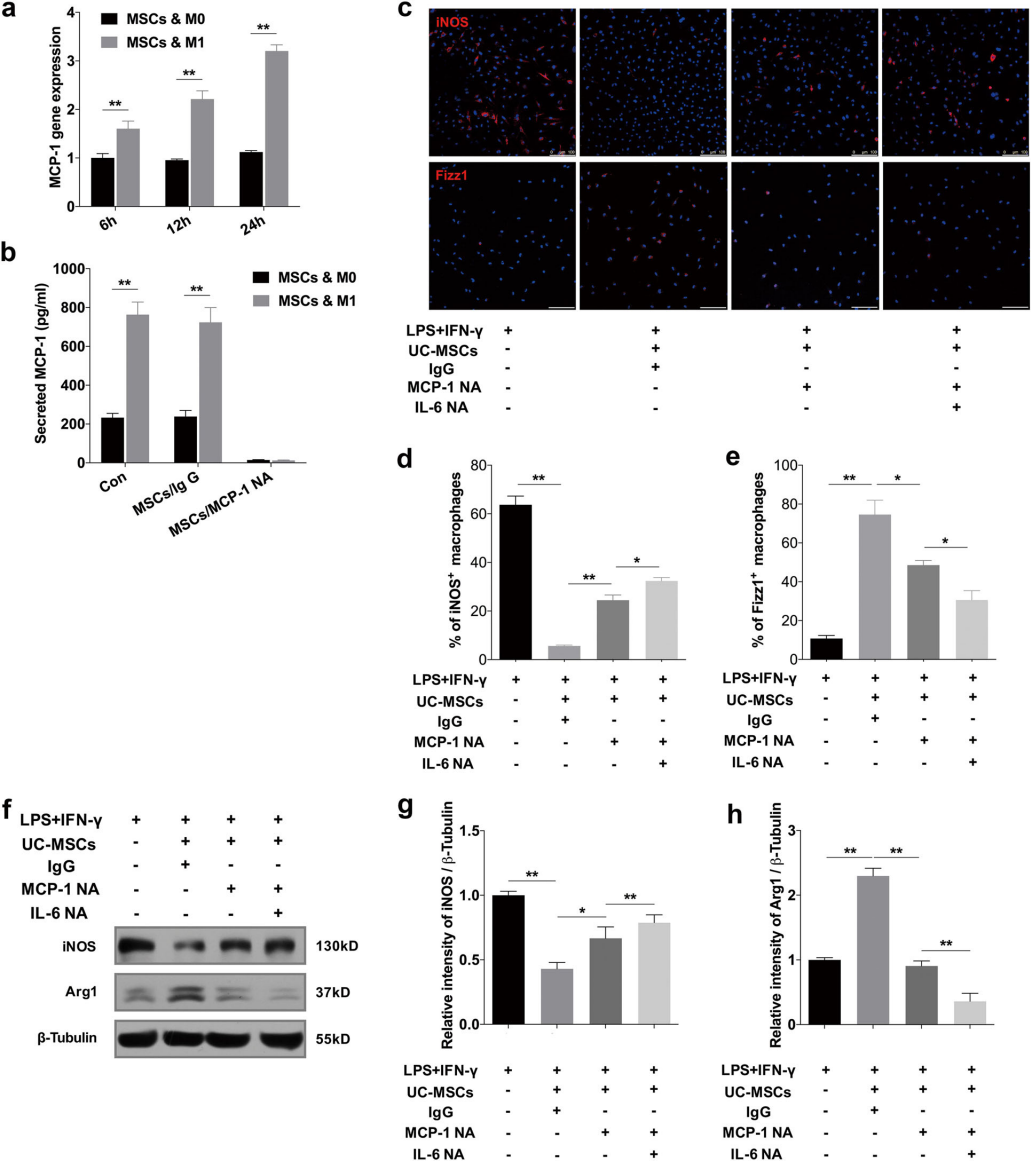
HUC-MSCs secreted MCP-1 to coordinate with IL-6 in regulating macrophage polarization. **a** Quantitative RT-PCR analysis of MCP-1 expression in hUC-MSCs. HUC-MSCs were cultured with M0 or M1 for 6, 12, 24 h. Results were presented relative to those of UC- MSCs cultured with M0, set as 1. **b** Secretory level of MCP-1 in medium of hUC-MSCs (Con), hUC-MSCs conditioned with IgG (MSCs/IgG) or hUC-MSCs conditioned with MCP-1 neutralizing antibody (NA) (MSCs/MCP-1 NA). HUC-MSCs were cultured with M0 or with M1. **c** Immunofluorescence of iNOS^+^-BMDMs and Fizz1^+^-PMs in stimulation with LPS and IFN-γ cultured alone, with hUC-MSCs conditioned with IgG, hUC-MSCs conditioned with MCP-1 neutralizing antibody (NA), or hUC-MSCs conditioned with MCP-1 neutralizing antibody (NA) plus IL-6 NA. Scale bar, 100 μm. Quantification of iNOS^+^ BMDMs and Fizz1^+^ PMs were presented in **d** and **e**. **f** Immunoblotting analysis of iNOS and Arg1 in BMDMs of the same four groups mentioned in **c**. Relative protein level is quantified by ratio of iNOS to β-tubulin **g** and that of Arg1 to β-tubulin **h**. Values are means ± SD of three individual experiments, *p < 0.05, **p < 0.01

Considering there is evidence demonstrating the role of MCP-1 in coordinating with IL-6 in inducing M2-type macrophage polarization^32^, we further investigated the expression of MCP-1 in hUC-MSCs. It was found that the mRNA expression of MCP-1 in hUC-MSCs significantly increased after co-cultured with M1 BMDMs (Fig. 8a). Accordingly, the level of MCP-1 secreted by hUC-MSCs rose to nearly 800 pg/ml in the cultured medium (Fig. 8b). To verify the role of MCP-1 in hUC-MSCs, MCP-1 neutralizing antibody (NA) was added in the medium to block MCP-1 secreted by hUC-MSCs. IgG served as the control condition. After neutralization, the concentration of MCP-1 in supernatant was reduced to lower than 15 pg/ml (Fig. 8b). With this extreme low level of MCP-1, we found that the expression of iNOS greatly increased, while that of Fizz1 and Arg1 significantly decreased at protein level according to the immunofluorescence (Fig. 8c–e) and immunoblotting analysis (Fig. 8f–h). Together with MCP-1 NA, we also neutralized IL-6 by IL-6 NA, after which the concentration of IL-6 was reduced to less than 5 pg/ml. The results showed that expression of iNOS was further increased while expression of Fizz1 and Arg1 was further decreased, which indicated that simultaneous blocking of MCP-1 and IL-6 led to greater inhibition to the modulatory role of hUC-MSCs (Fig. 8c–h). These data suggested that MCP-1 and IL-6 secreted by hUC-MSCs could work in accordance to direct macrophage polarization from M1 to M2 state.

## Discussion

Macrophages within islets are known to influence β-cell function, proliferation, and apoptosis. Under normal physiological conditions, macrophages are constitutively present within islets, where they provide a suitable microenvironment for β-cell development and the maintenance of islet homeostasis^33,34^. However, during the progression of T2D, fatty acids stimulate pancreatic β-cells to produce chemokines that promote intra-islet accumulation of M1 macrophages^5,25^, and together with other TLR2/6 and TLR4 ligands stimulate the secretion of proinflammatory factors such as IL-1β from macrophages^35^. As IL-1β receptor is abundantly expressed on β-cells^36^, the high concentration of IL-1β subsequently suppresses both proliferation of β-cells and their secretion of insulin^37^, even lead to β-cell apoptosis^38,39^. However, emerging evidences have revealed a role for M2 macrophages during β-cell protection, repair, and regeneration. Upon helminth infection, an important TH2 response is induced, with an alteration in macrophage phenotype from M1 to M2^40^. The helminth infection increases islet infiltration of M2 macrophages, which results in reduced insulitis and β-cell loss, thus preventing the onset of diabetes in HFD^41^, non-obesity diabetes (NOD)^42^, and multiple low dose STZ-treated mice^43^. Another report shows that M2 macrophages recruited to the pancreas after partial duct ligation (PDL) promotes β-cell proliferation by secreting TGFβ and epidermal growth factor^44^. In our study, we found that HFD and STZ not only induced intra-islet accumulation of M1 macrophages, but also elicited a slight increase in the number of M2 macrophages. This was similar to the finding observed in db/db mice where the increased M2 macrophages were suggested to be contributed by a systemic shift from M1 macrophages^45^. However, the moderately increased M2 macrophages seem not sufficient to reverse the proinflammatory microenvironment. After hUC-MSCs infusion, a significantly increased proportion of M2 macrophages occurred in the pancreas, accompanied by a downregulation in that of M1 macrophages and reduced expression of proinflammatory mediators. After knockdown of IL-6, the effects of hUC-MSCs on macrophage phenotype modulation was dampened, meanwhile, therapeutic effects of hUC-MSCs on islets protection was greatly impaired.

In our study, we found that MCP-1 expression in islets was remarkably reduced after hUC-MSCs infusion, but the number of macrophages in islets did not change significantly. This may be due to that macrophage recruitment mainly occurred in the early phase of islets damage, as the number of intra-islet macrophages of T2D group changed little at third and fifth week following the STZ injection compared with that at second week after the injection (data not shown). Besides, in addition to MCP-1, macrophages have other chemotactic mechanisms. Studies have shown that macrophages trafficking from the bone marrow mainly depend on the chemokine receptor CCR2 and its ligand MCP-1^46^, while migration of spleen leukocyte is mediated by angiotensin II (AT-2) released from impaired tissues and AT-2 receptor-1α (ATR-1α) expressed on monocytes and macrophages^47^. In our previous study, we found that a large number of hUC-MSCs homed to spleen after infusion, accompanied with increased M2-like macrophages in the spleen (data not shown). These M2-like macrophages are likely to mobilized in large numbers to the site of tissue injury^47–49^, and in this case, the injured tissue could be the pancreas. Another possibility could be local macrophage proliferation in the injured pancreas. In steady state^50^ and PDL^51^, resident macrophages were reported to proliferate in a macrophage colony-stimulating factor (M-CSF)-dependent manner. And MSCs have been demonstrated to promote proliferation of macrophages via secreting M-CSF^52^.

IL-6 and MCP-1 are often considered to be pro-inflammatory cytokines associated with progression of T2D^53,54^. However, emerging studies have suggested that these two factors can modulate macrophages toward an M2-like state. MCP-1, together with Siglec-9, are indicated to improve acute liver failure by altering macrophage polarity in rats^55^. Our own work demonstrated that IL-6 secreted by hUC-MSCs could elicit macrophages into an anti-inflammatory phenotype in adipose tissue, thereby alleviating insulin resistance in T2D rats^22^. The different inflammatory properties of these two cytokines revealed in different studies may be related to diverse concentration, utility duration, and complex mechanisms on different cell types. In addition, whether the increased MCP-1 and IL-6 in inflammation-related diseases is the cause or manifestation of tissue injury still needs further exploration.

In conclusion, hUC-MSCs treatment restored islets function in T2D mice, and this effect was partially attributed to suppressing inflammation and inducing M2 macrophages polarization via the secreted MCP-1 and IL-6 from hUC-MSCs. These data may provide new clues for searching for the target of protecting islet β-cell function, and provide a theoretical basis for hUC-MSCs use in the clinic as a treatment.

## Materials and Methods

### Isolation and culture of hUC-MSCs

HUC-MSCs were isolated from human umbilical cords freshly obtained from women who gave birth in the Chinese PLA General Hospital. All of the subjects provided informed consent. The characteristics of hUC-MSCs were identified by phenotypes and the potential to differentiate into adipocytes and osteoblasts^22^. Protocols were approved by the Ethics Committee of the Chinese PLA General Hospital. For selective knockdown of IL-6, hUC-MSCs were transfected with siRNA for IL-6 (IL-6 siRNA) (GenePharma, Suzhou, China) using Lipofectamine RNAiMAX (Invitrogen, NY, USA). hUC-MSCs transfected with scrambled siRNA (scr siRNA) (GenePharma, Suzhou, China) served as control. The protocol and sequences for IL-6 siRNA and scr siRNA were described previously^22^. For neutralization experiments, 50ug/ml IL-6 NA (R&D Systems, Minneapolis, MN, USA) and/or 5 μg/ml MCP-1 NA (R&D Systems) were added to the cultured medium when hUC-MSCs were co-culturing with M1 BMDMs. To confirm successful knockdown or blockade of IL-6 and MCP-1, RNA and/or cultured medium of hUC-MSCs were obtained to measure the expression of IL-6 and/or MCP-1 by quantitative real-time reverse transcriptase polymerase chain reaction (RT-PCR) or enzyme-linked immunosorbent assay (ELISA).

### T2D induction and treatment

8-week-old male C57BL/6J mice purchased from the Chinese PLA General Hospital were fed with HFD (60% fat, Research Diets, New Brunswick, NJ) or a normal chow diet (NCD) for 12 weeks. To achieve T2D model, a single dose of 100 mg/kg STZ (Sigma-Aldrich, St. Louis, MO) dissolved in 0.05 M citrate buffer (pH 4.5), was intra-peritoneally injected to HFD-fed mice. Blood glucose levels were determined by monitoring tail capillary blood glucose levels with a glucometer (Yuwell, Jiangsu, China). One week after STZ administration, intraperitoneal glucose tolerance tests (IPGTTs) and insulin-tolerance tests (IPITTs) were applied to confirm the T2D model. For IPGTT or IPITT, overnight fasted mice were intra-peritoneally injected with glucose (1.5 g/kg) or insulin (1 U/kg), glucose levels of which were determined by tail blood samples taken every 30 min up to 120 min. The established T2D mice were then randomly treated with an infusion of 1 × 10^6^ hUC-MSCs suspended in 0.2 ml phosphate-buffered saline (PBS) (referred as the hUC-MSCs group) through the tail vein or with an infusion of 0.2 ml PBS alone (referred as the T2D group). In order to demonstrate the homing of hUC-MSCs in pancreas, a random collection of T2D mice were treated with hUC-MSCs labeled with chloromethyl-benzamidodialkylcarbocyanine (CM-Dil, Life technologies, Eugene, Oregon, USA) in advance according to the manufacturer’s instruction. One week later, IPGTT and IPITT were performed again to assess the therapeutic effects of hUC-MSCs. Blood plasma was obtained. Fasting blood glucose (FBG) was detected in the Biochemistry Department of the Chinese PLA General Hospital. Fasting blood insulin (FBI) was measured by commercial ELISA kit (Mercodia, Uppsala, Sweden). The homeostatic model assessment for insulin resistance (HOMA-IR) and for β-cells (HOMA- β) were calculated by the following equation: (FBG [in mmol/l] × FBI [in mIU/l])/22.5, 20× FBI [in mIU/l]/(FBG [in mmol/l]−3.5) (%). All in vivo experimental procedures were reviewed and approved by the medical ethics committee of the Chinese PLA General Hospital.

### Generation and stimulation of macrophages

Peritoneal macrophages (PM) were achieved from 6-week-old C57BL/6J male mice by peritoneal lavage with 2 ml RMPI 1640 (Gibco, Grand Island, NY, USA), and then cultured in RMPI 1640 supplemented with 10% fetal bovine serum (Gibco) and 1% penicillin streptomycin (Gibco). Identification of PMs were done by anti-F4/80 (1:50, Life technologies, Frederick, USA) immunofluorescence. BMDMs were isolated from the femur and tibia of 6-week-old C57BL/6J male mice and then cultured in RMPI 1640 supplemented with 100 ng/ml M-CSF (R&D system), 10% fetal bovine serum and 1% penicillin streptomycin. Identification of BMDMs were done by anti-F4/80 (eBioscience, San Diego, CA, USA, Clone:BM8) flow cytometry. Lipopolysaccharides (100 ng/ml, LPS, Sigma-Aldrich) and interferon-γ (50 ng/ml, IFN-γ, R&D Systems) were added in the medium to stimulate the two types of macrophages for 24 h. The macrophages were then cultured with 5 × 10^4^ hUC-MSCs in a trans-well system for another 24 h.

The human monocytic cell line THP-1 was purchased from the American Type Culture Collection (ATCC, Manassas, VA, USA). THP-1 cells were cultured in RPMI 1640 medium supplemented with 10% FBS and 1% penicillin streptomycin at a density of 3 × 10^5^–6 × 10^5^ cells/ml as recommended by the ATCC. The differentiation was induced by treatment with phorbol 12-myristate 13-acetate (PMA, 160 ng/ml, Sigma). After 24 h, the non-adherent cells were removed with three rinses with PBS. Adherent cells were further incubated with fresh medium containing LPS(100 ng/ml) and IFN-γ(50 ng/ml, Prime Gene Bio-Tech, Shanghai, China) or combined cytokines including IAPP (0.1 μM/ml, Prime Gene Bio-Tech), CCL2 (10 ng/ml, Prime Gene Bio-Tech), CXCL1 (10 ng/ml, Prime Gene Bio-Tech) and IL-1β (10 ng/ml, Prime Gene Bio-Tech) for 24 h. After that, conditioned medium was removed and incubated with fresh medium alone or together with hUC-MSCs by trans-well for another 36 h.

### Flow cytometric analysis

Freshly obtained pancreases were rinsed in PBS, and then minced into little pieces. Tissues were digested in Hank’s Balanced Salt Solutions (HBSS) (TBD, Tianjin, China) with 2 mg/ml V collagenase (Sigma-Aldrich) for 10–15 min at 37 ℃ with shaking. D-HBSS (TBD, Tianjin, China) was added to terminate the digestion and the cell suspensions were filtered through a 400-mesh metal filter and centrifuged at 1500 rpm for 5 min. The pellets were then incubated with PE-conjugated anti F4/80 (eBioscience), FITC-conjugated anti-CD206 (BioLegend, San Diego, CA, USA Clone: C068C2) and APC-conjugated anti-CD11c (Miltenyi Biotec, Bergisch Gladbach, Germany) antibodies for flow cytometry analysis.

Freshly harvested BMDM were washed twice and then stained with PE-conjugated anti F4/80 (eBioscience) antibody for flow cytometry analysis. The THP-1 cells were washed twice before incubated with fix&perm and perm (BD), and them stained with PE-conjugated anti CXCL-10 (eBioscience) antibody for flow cytometry analysis.

### Immunofluorescence staining

Mice were sacrificed at indicated time points, for which 1% pentobarbital sodium (50 mg/kg) was intra-peritoneally injected, and then 10 ml PBS was perfused through the left ventricle followed with 15 ml 4% paraformaldehyde. After the perfusion, pancreases were isolated and incubated in 30% sucrose/PB overnight. The tissues were then embedded with optimal cutting temperature compound (OCT, Sakura, Finetek, USA) and made into frozen sections at 6 μm. For immunofluorescence analysis, frozen sections were blocked in bovine serum albumin and incubated at 4 °C overnight with primary antibodies of insulin (1/200, guinea pig, Abcam, San Francisco, USA), Glucogon (1/2000, mouse, Abcam), TUNEL (Roche, Mannheim, Germany), Ki67 (1:300, rabbit, Abcam), PDX-1 (1:300, rabbit, Cell Signaling Technology), MafA(1:200, rabbit, Bethyl Laboratories), MCP-1 (1:100, rabbit, Santa Cruz Biotechnology), IL-1β (1:100, rabbit, Abcam), F4/80 (1:50, mouse, Life Technologies), CD11c (1:100, mouse, Abcam), Fizz1 (1:100, rabbit, Abcam,), iNOS (1:250, rabbit, Abcam), CD163 (1:200, mouse, Bio-rad, CA, USA), ARG1 (1:100, rabbit, Abcam), VEGF (1:100, rabbit, ZENBIO, China), IL-6 (1:100, rabbit, Abcam), CD206 (1:100, rabbit, Abcam), then with Alexa Fluor 488/594-conjugated secondary antibodies (1: 500, Life technologies) at room temperature for 2 h. The nuclei were visualized with 4,6-diamidino-2-phenylindole (DAPI) (Sigma-Aldrich). The sections were observed under laser scanning confocal microscope (Leica, Wetzlar, Germany). PMs and THP-1 cells were plated onto glass coverslips and fixed with 4% paraformaldehyde for 15 min. The following steps were performed as described above.

### Cytokine profile

HUC-MSCs were co-cultured with PM or BMDM for 24 h, after which the culture supernatant was harvested. The secretion of IL-6 and MCP-1 from hUC-MSCs was measured using commercial ELISA kits from NeoBioscience, Shenzhen, China. The secretion of IL-1β, Tnf-α, and IL-4 from BMDM were quantified by AimPlex™ assay kits (QuantoBio, Beijing, China) according to manufacturer instruction, together with mouse blood serum MCP-1, IL-1β, IL-4, IL-6, IL-10, and TNF-α. Levels of IL-1β and IL-10 in the supernatant of THP-1 cells were also evaluated by ELISA kits from NeoBioscience, Shenzhen, China.

### Western blot

Total protein was extracted from BMDM, and the experiment was carried out as described previously^56^. The primary antibodies were iNOS (1:1000, rabbit, Abcam), Arg1 (1:1000, rabbit, Abcam), and β-Tubulin (1:2000, mouse, ZSGB-Bio, Beijing, China). The secondary antibodies were goat anti-rabbit, and rabbit anti-mouse IgG horse radish peroxidase (HRP) (1:3000, mouse, ZSGB-Bio). The blots were analyzed using Image J software (NIH, Bethesda, MD, USA).

### Quantitative real-time reverse transcriptase polymerase chain reaction

Total RNA samples were extracted from BMDM, THP-1 cells, and hUC-MSCs using TRIzol reagent (Life Technologies), and then reversely transcribed to single-stranded cDNA with a reverse transcription kit (Thermo Scientific, CA, USA) according to the instructions. Real-time polymerase chain reaction (RT-PCR) was performed on ABI Prism thermal cycler model StepOnePlus (Applied Biosystems, CA, USA) using a SYBR Green PCR master mix (Applied Bio-systems). The thermal cycling program was 94 ℃ for 3 min, followed by 94 ℃ for 30 s, 60 ℃ for 30 s and 72 ℃ for 30 s for 40 cycles. Melting curve analysis was included to ensure primer specificity. Gapdh was used as the internal control. The primers were listed in Supplementary Table 1 and 2.

### Statistical analysis

All values are presented as means ± SD from at least three independent samples. Differences between means were assessed using a Student’s t test or Chi-square test when required. A two-tailed *p* < 0.05 was considered statistically significant. All analyses were performed using SPSS version 14.0.1 (SPSS Inc., IBM).

## Electronic supplementary material

SUPPLEMENTAL MATERIAL
