# Supplementary material for: Human umbilical cord-derived mesenchymal stem cells direct macrophage polarization to alleviate pancreatic islets dysfunction in type 2 diabetic mice
Source: Cell Death Dis. 2018 Jul 9;9(7):760. doi: 10.1038/s41419-018-0801-9 (PMC6037817; doi:10.1038/s41419-018-0801-9)
Supplement: Supplementary file 1 — SUPPLEMENTAL MATERIAL [file 41419_2018_801_MOESM1_ESM.docx]

**Supplementary Information**

**
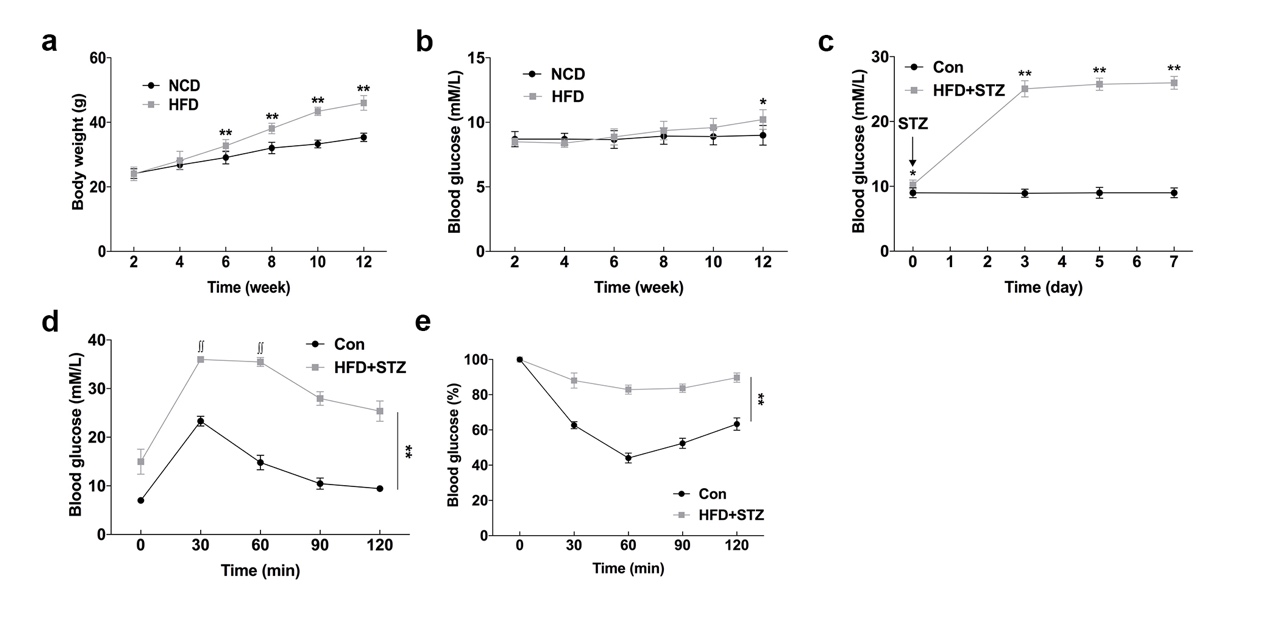
**

**Supplementary Fig 1 Induction of T2D mouse model.** T2D mouse model was induced by a combination of 12-week HFD and a single introperitoneal injection of low-dose STZ. Body weight (a) and random blood glucose (b) of the mice were measured every 2 weeks. After STZ injection, blood glucose (c) was consecutively detected. One week after STZ injection, IPGTT (d) and IPITT (e) were performed to confirm the establishment of T2D mouse model. ∫∫, blood glucose level exceeded the maximum (36 mmol/L) of the glucometer. For IPITT (e), results were presented relative to initial blood glucose levels. The results were presented as the means ± SD. **p*<0.05, ***p*<0.01.


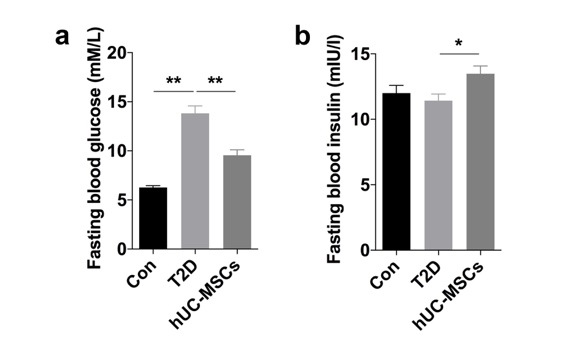


**Supplementary Fig 2 HUC-MSCs administration improved glucose homeostasis in T2D mice.** The levels of fasting blood glucose (a) and fasting blood insulin (b) of the three groups. The results were presented as the means ± SD. **p*<0.05, ***p*<0.01.


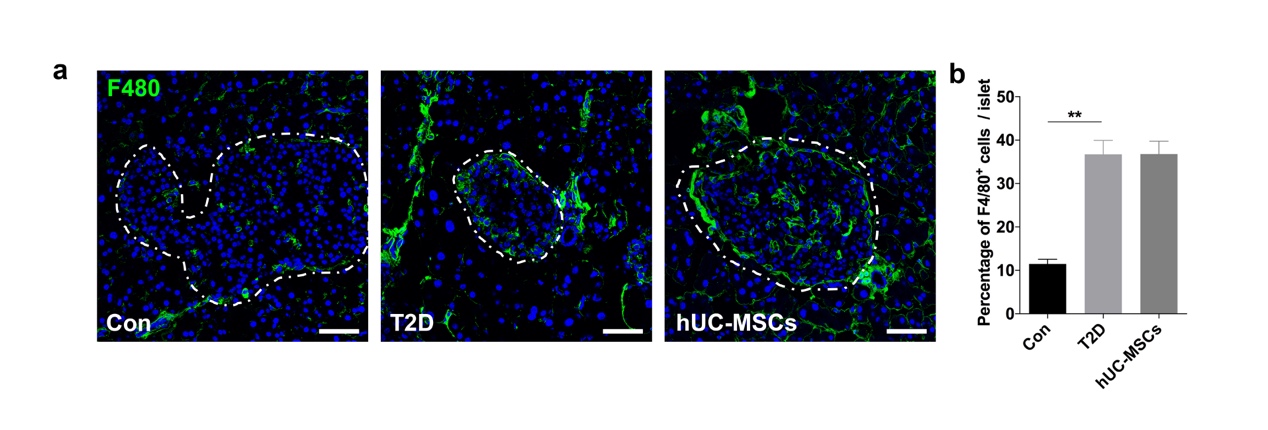


**Supplementary Fig 3 UC-MSCs administration did not achieve significant effect on the amount of macrophages in pancreas.** (a) Photomicrographs of representative islets stained with anti-F4/80 (green) antibody from the Con, the T2D and the hUC-MSCs group. The dotted line circled areas are pancreatic islets. Scale bar, 50μm. Quantification of F4/80^+^ cells shown in (b) was determined by evaluating islets from at least 6 sections of each group. The results were presented as the means ± SD. **p<0.01.


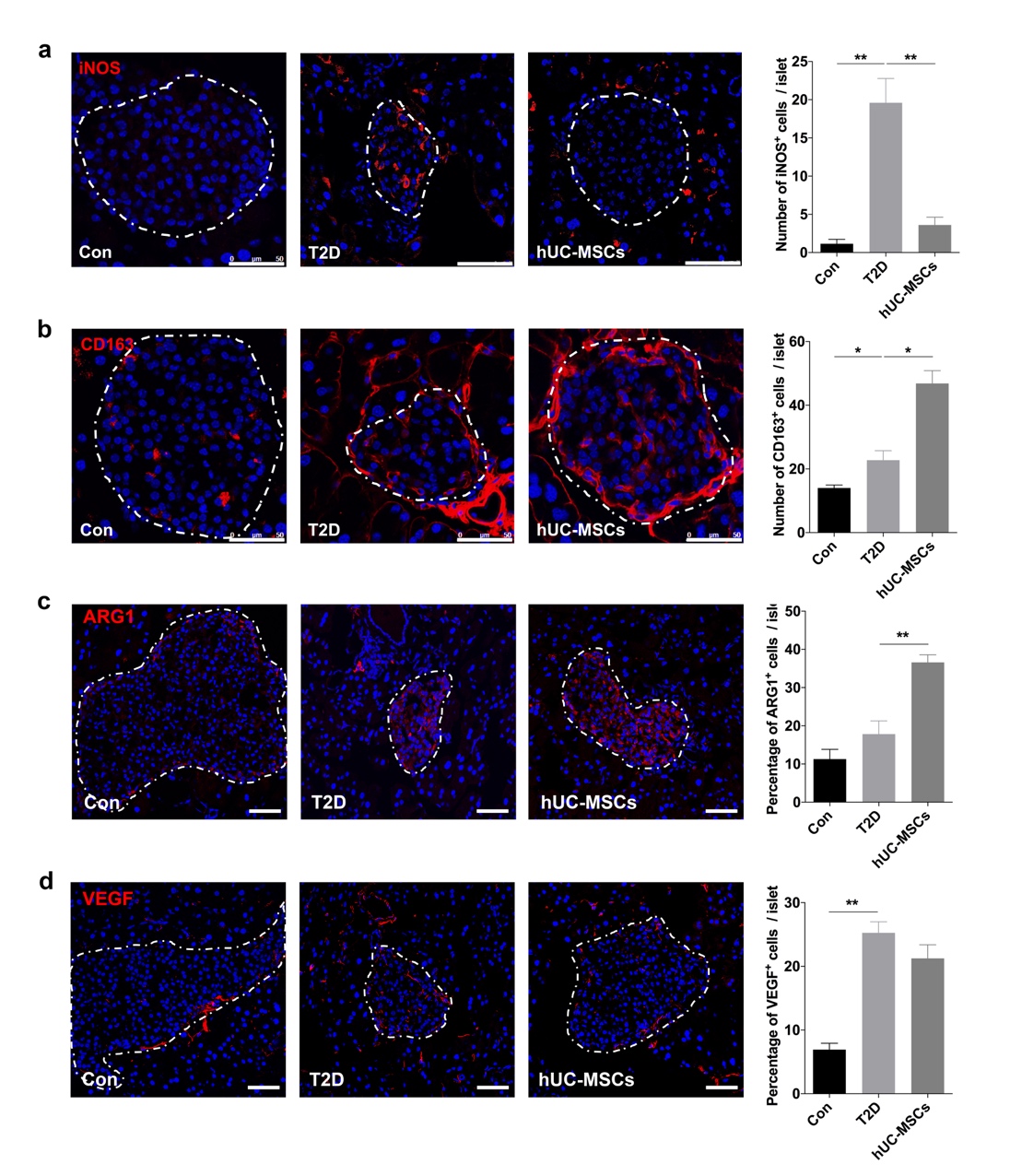


**Supplement Fig 4 HUC-MSCs infusion induced M2 macrophage polarization in pancreatic islets.** Photomicrographs of representative islets from the Con, the T2D and the hUC-MSCs group stained with anti-iNOS (a), anti-CD163 (b), anti-ARG1 (c) and anti-VEGF (d) antibodies. The dotted line circled areas are pancreatic islets. Scale bar, 50μm. The results were presented as the means ± SD. *p<0.05, **p<0.01. Abbreviations: iNOS, inducible nitric oxide synthase. ARG1, arginase-1. VEGF, vascular endothelial growth factor.


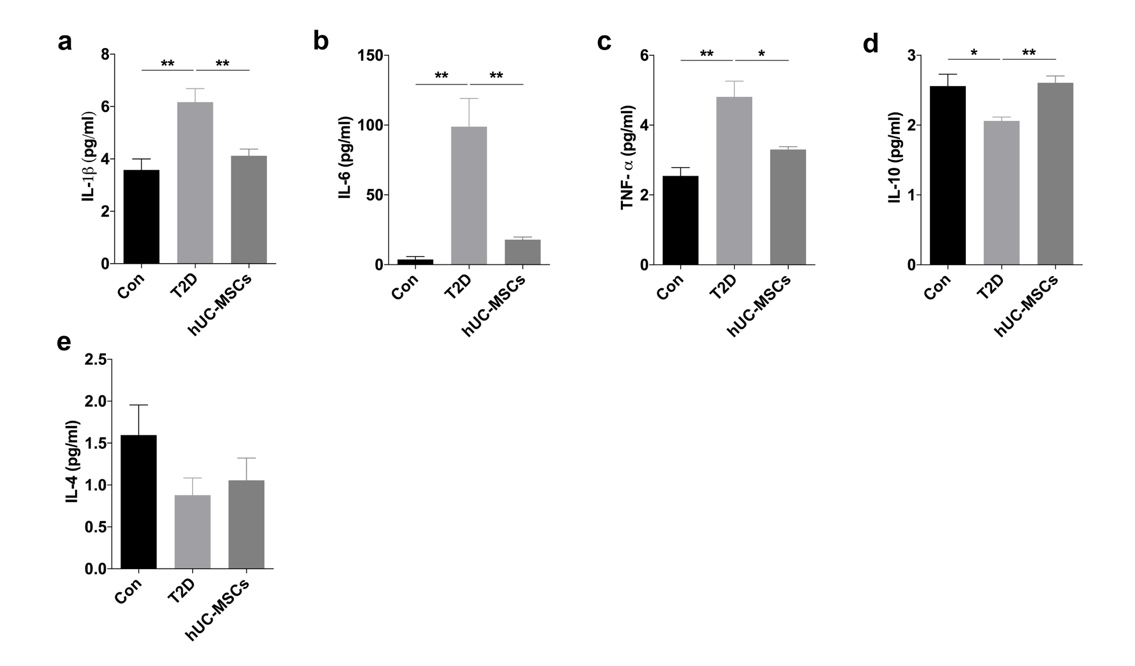


**Supplement Fig 5 HUC-MSCs infusion effectively alleviate the chronic inflammation in T2D individuals.** Serum levels of IL-1β (a), IL-6 (b), TNF-α (c), IL-10 (d) and IL-4 (e) were measured by AimPlex™ assay. The results were presented as the means ± SD. *p<0.05, **p<0.01. Abbreviations: IL, interleukin. TNF-α, tumor necrosis factor-α.

**
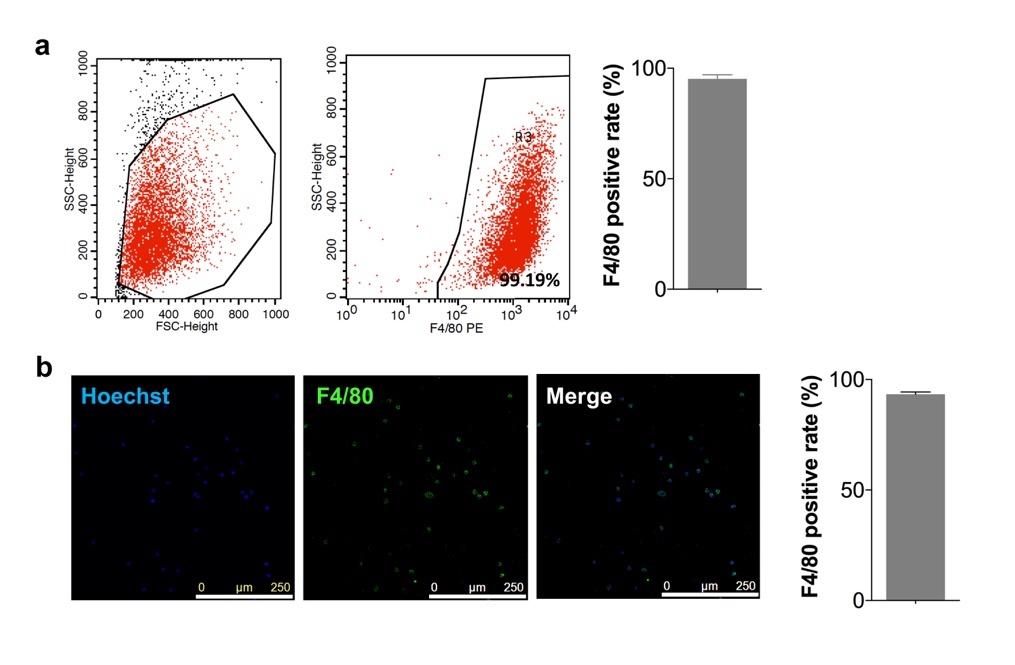
**

**Supplement Fig 6 Identification of BMDMs and PMs.** BMDMs were isolated and cultured in RMPI 1640 supplemented with 100 ng/ml M-CSF. (a) Flow cytometry analyses showed that the positive rate of F4/80 in the bone marrow derived cells was over 95%. (b) PMs were achieved by peritoneal lavage and cultured in RMPI 1640. Immunofluorescence detection revealed that about 93% of the cells were F4/80 positive. Scale bar, 250μm. Results are means ± SD. of three separate experiments. Abbreviations: BMDMs, bone marrow derived macrophages. M-CSF, macrophage colony-stimulating factor. PMs, peritoneal macrophages.


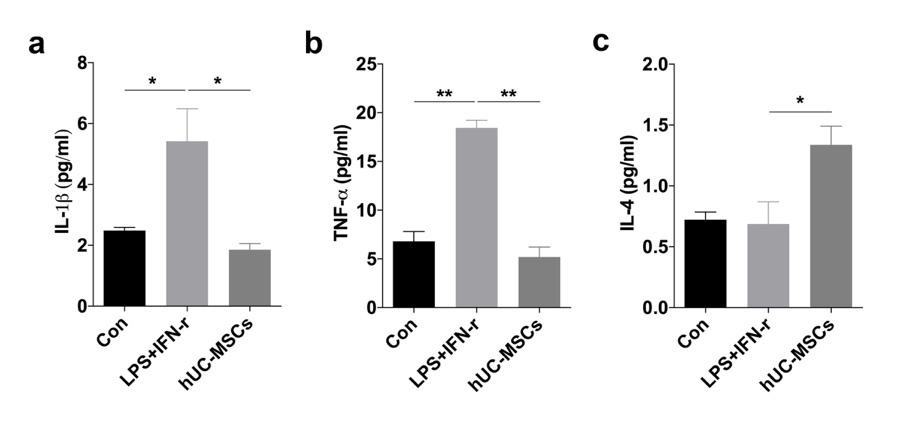


**Supplement Fig 7 HUC-MSCs inhibited the secretion of pro-inflammatory mediators and increased that of anti-inflammatory cytokines from BMDMs.** The secretion levels of IL-1β (a), TNF-α (b) and IL-4 (c) in the supernatant of the BMDMs were measured by AimPlex multiplex assay. Results are means ± SD. of three replicates from four separate experiments. *p<0.05, **p<0.01. Abbreviations: LPS, lipopolysaccharides. IFN- γ, interferon- γ. IL, interleukin.


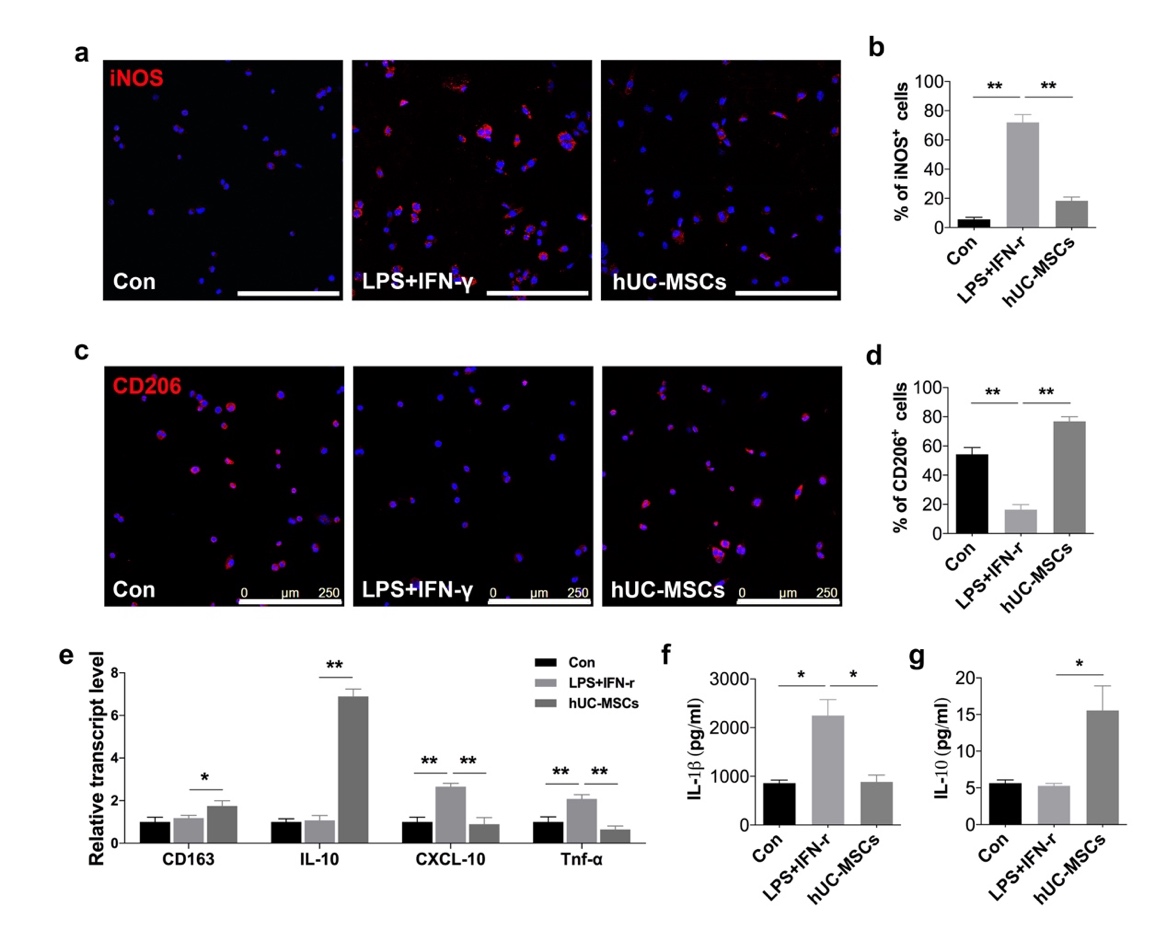


**Supplement Fig 8 HUC-MSCs suppressed M1 phenotype and induced M2 phenotype in THP-1** **cells stimulated by LPS and IFN- γ.** THP-1 cells were cultured in RPMI 1640 medium supplemented with 10 % FBS and 1% penicillin streptomycin. The differentiation was induced by treatment with phorbol 12-myristate 13-acetate (PMA, 160 ng/ ml, Sigma). After 24 hours, the non-adherent cells were removed by three rinses with PBS. Adherent cells were further incubated with fresh medium (Con group) or medium containing LPS(100ng/ml) and IFN-γ(50ng/ml) for 24 hours. After that, conditioned medium was removed and incubated with fresh medium alone (LPS+IFN-γ group) or together with hUC-MSCs by transwell (hUC-MSCs group) for another 36 hours. Images are representatives of immunofluorescence results of iNOS^+^-cells (a) and CD206^+^-cells in the three groups. Scale bar, 250μm. Quantification of iNOS^+^-cells and CD206^+^-cells presented in (b) and (d) were determined by evaluating at least 5 random fields of each section. (e) Quantitative RT-PCR analysis of gene expression in THP-1 cells from the three groups. Results are presented relative to those of the control group, set as 1. The secretion levels of IL-1β (f) and IL-10 (g) in the supernatant were measured by ELISA. Values are means ± SD. of three individual experiments. *p<0.05, **p<0.01.


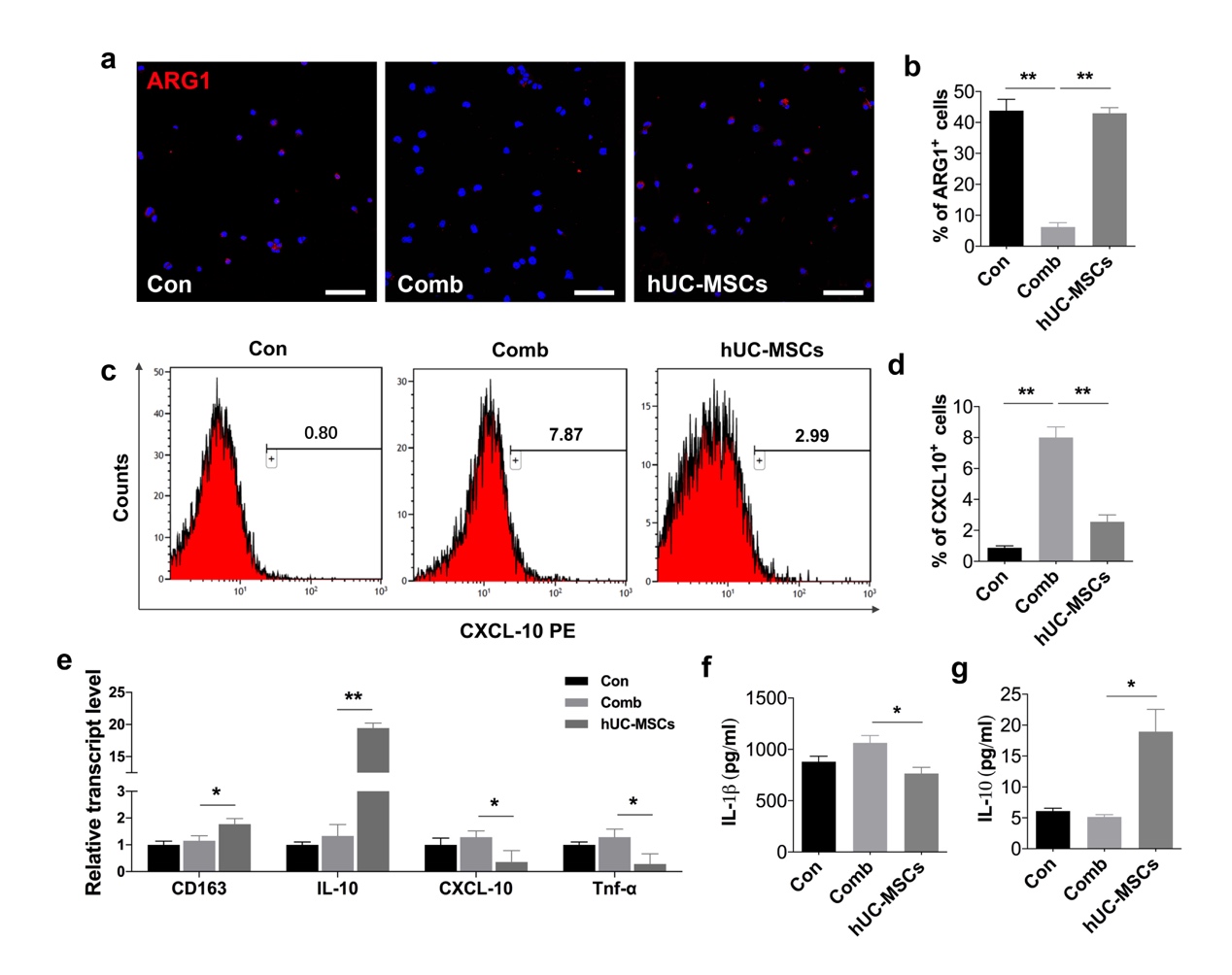


**Supplement Fig 9 HUC-MSCs suppressed M1 phenotype and induced M2 phenotype in THP-1** **cells stimulated by combined cytokines.** The THP-1 cells were cultured in PMA conditioned RPMI-1640 for 24 hours. After that, adherent cells were further incubated in fresh medium without (Con group) or containing IAPP (0.1μM/ml), CCL2 (10ng/ml), CXCL1 (10ng/ml) and IL-1β (10ng/ml) for another 24 hours and then cultured in fresh medium alone (Comb group) or together with hUC-MSCs (hUC-MSCs group) for 36 hours. (a) Images are representatives of immunofluorescence results of ARG1^+^-cells in the three groups. Scale bar, 100μm. Quantification of ARG1^+^-cells presented in (b) was determined by evaluating at least 5 random fields of each section. (c) Flow cytometry analysis was used to detect the proportion of CXCL-10^+^ cells in each group, quantification of which was shown in (d). (e) Quantitative RT-PCR analysis of gene expression in THP-1 cells from the three groups. Results are presented relative to those of the control group, set as 1. The secretion levels of IL-1β (f) and IL-10 (g) in the supernatant were measured by ELISA. Values are means ± SD. of three individual experiments. *p<0.05, **p<0.01. Abbreviations: IAPP, islet amyloid polypeptide.


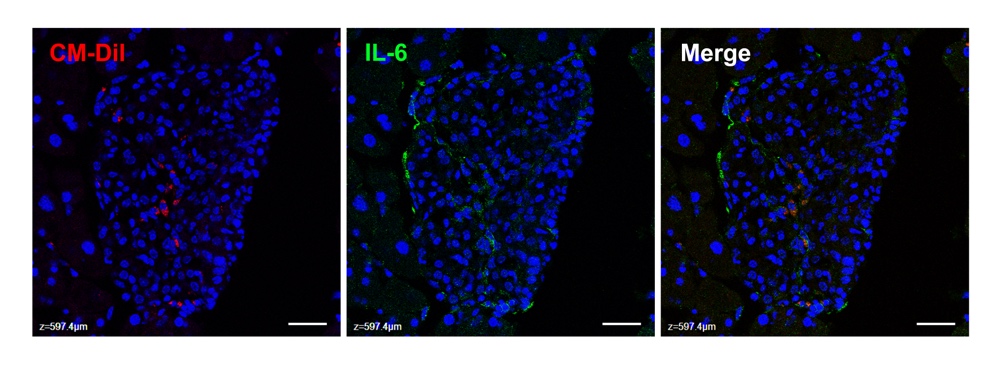


**Supplement Fig 10 Co-staining of IL-6 and hUC-MSCs in pancreatic islets.** T2D mice were infused with CM-Dil pretreated hUC-MSCs. Five days after the infusion, the mice were sacrificed, the pancreas were obtained and made into frozen sections. IL-6 immunofluorescent antibody was used to detect the co-localization of IL-6 and CM-Dil. Scale bar, 100μm.


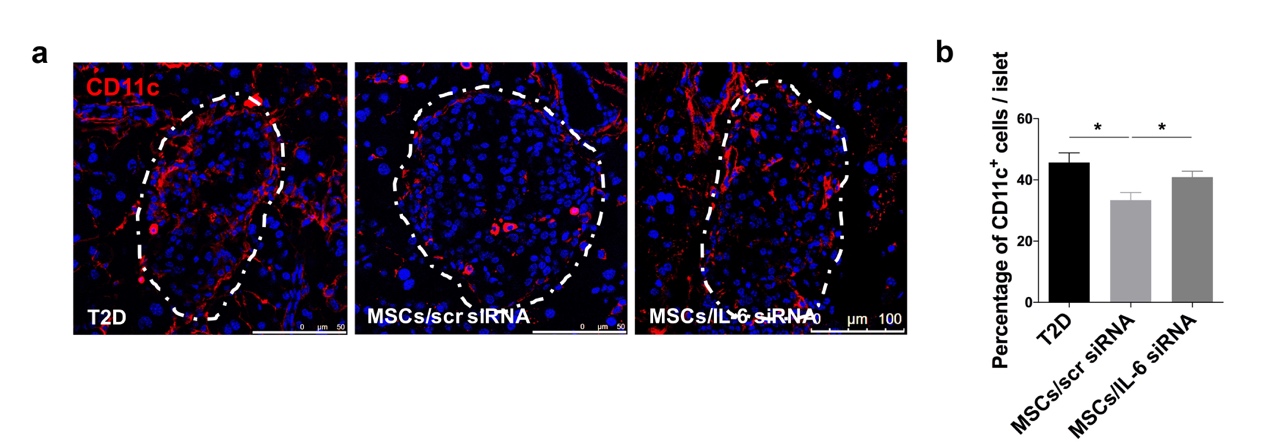


**Supplement Fig 11 HUC-MSCs modulated macrophage polarization via secretion of IL-6 in vivo.** (a) Photomicrographs of representative islets stained with anti-CD11c (red) antibody from the T2D mice without (T2D group) or with the administration of hUC-MSCs transfected with scrambled siRNA (MSCs/scr siRNA group) or hUC-MSCs transfected with IL-6 siRNA (MSCs/IL-6 siRNA group). The dotted line circled areas were pancreatic islets. Scale bar, 100μm. Quantifications of CD11c^+^ cells within islets (b) was determined by evaluating islets from at least 5 sections of each group. Values are means ± SD. *p<0.05.

**Supplementary Table 1 Primer sequences of target genes (mice)**

| Genes | Primer sequence (5’-3’) |
| --- | --- |
| Gapdh | For: ACTCCCACTCTTCCACCTTC |
|  | Rev: TCTTGCTCAGTGTCCTTGC |
| CD11c | For: ACGTCAGTACAAGGAGATGTTGGA |
|  | Rev: ATCCTATTGCAGAATGCTTCTTTACC |
| Nos2 | For: ACCTTGGTGAAGGGACTGAG |
|  | Rev: TCCGTTCTCTTGCAGTTGAC |
| IL-1β | For: TGGGCCTCAAAGGAAAGAAT |
|  | Rev: CAGGCTTGTGCTCTGCTTGT |
| Tnf-α | For: CCAGACCCTCACACTCAGATC |
|  | Rev: CACTTGGTGGTTTGCTACGAC |
| CD206 | For: TGATTACGAGCAGTGGAAGC |
|  | Rev: GTTCACCGTAAGCCCAATTT |
| Arg1 | For: AGACCACAGTCTGGCAGTTG |
|  | Rev: CCACCCAAATGACACATAGG |
| IL-4 | For: GGTCTCAACCCCCAGCTAGT |
|  | Rev: GCCGATGATCTCTCTCAAGTGAT |
| IL-10 | For: GCTCTTACTGACTGGCATGAG |
|  | Rev: CGCAGCTCTAGGAGCATGTG |
| Tgf-β | For: ATTCCTGGCGTTACCTTGG |
|  | Rev: AGCCCTGTATTCCGTCTCCT |

**Supplementary Table 2 Primer sequences of target genes (human)**

| Genes | Primer sequence (5’-3’) |
| --- | --- |
| Gapdh | For: GGAGCGAGATCCCTCCAAAAT |
|  | Rev: GGCTGTTGTCATACTTCTCATGG |
| IL-6 | For: GGTACATCCTCGACGGCATCT |
|  | Rev: GTGCCTCTTTGCTGCTTTCAC |
| Ido | For: GCCAGCTTCGAGAAAGAGTTG |
|  | Rev: ATCCCAGAACTAGACGTGCAA |
| Tgf-β | For: CCCTGGACACCAACTATTGC |
|  | Rev: TGCGGAAGTCAATGTACAGC |
| Tsg6 | For: GCTAGAGGCAGCCAGAAAAA |
|  | Rev: ATCCAACTCTGCCCTTAGCC |
| Pge2 | For: CCCTGGACACCAACTATTGC |
|  | Rev: TGCGGAAGTCAATGTACAGC |
| MCP-1 | For: ATGCAATCAATGCCCCAGTC |
|  | Rev: TGCAGATTCTTGGGTTGTGG |
| CD163 | For: CCAACAAGATGCTGGAGTGAC |
|  | Rev: TGACAGCACTTCCACATTCAAG |
| IL-10 | For: GGGAGAACCTGAAGACCCTC |
|  | Rev: ATAGAGTCGCCACCCTGATG |
| CXCL10 | For: ACTGTACGCTGTACCTGCATC |
|  | Rev: TGCAGATTCTTGGGTTGTGG |
| IL-1β | For: TTCCTGTTGTCTACACCAATGC |
|  | Rev: CGGGCTTTAAGTGAGTAGGAGA |
